# Biodiversity within phytoplankton-associated microbiomes regulates host physiology, host community ecology, and nutrient cycling

**DOI:** 10.1128/msystems.01462-24

**Published:** 2025-01-28

**Authors:** Jonathan R. Dickey, Nikki M. Mercer, Mirte C. M. Kuijpers, Ruben Props, Sara L. Jackrel

**Affiliations:** 1 Department of Ecology, Behavior and Evolution, University of California San Diego School of Biological Sciences, La Jolla, California, USA; 2 KYTOS BV, Zwijnaarde, Belgium; Boise State University, Boise, Idaho, USA

**Keywords:** host-microbiome, microbial biodiversity, aquatic ecology, ecosystem function, climate warming, phytoplankton, algal-bacterial interactions

## Abstract

Biological diversity is declining across the tree of life, including among prokaryotes. With the increasing awareness of host-associated microbes as potential regulators of eukaryotic host physiology, behavior, and ecology, it is important to understand the implications of declining diversity within host microbiomes on host fitness, ecology, and ecosystem function. We used phytoplankton and their associated environmental microbiomes as model systems to test the independent and interactive effects of declining microbiome diversity with and without other stressors often caused by human activity—elevated temperature and altered nutrient availability. We found effects of low microbiome diversity on host physiology, phytoplankton community dynamics, and nutrient cycling. Low microbiome diversity caused greater host cellular stress, as indicated by elevated δ^13^C and δ^15^N. Microbiome diversity also significantly affected host cell morphological metrics, likely as a consequence of this effect on cell stress. Despite causing greater host cellular stress, the effects of low microbiome diversity on host community ecology included elevated phytoplankton community diversity and biomass. The diversity of these host-associated microbes also had cascading implications on ecosystem nutrient cycling, where lower microbiome diversity caused a depletion of total dissolved N and P in the environment. The magnitude of these effects, caused by microbiome diversity, was greatest among nutrient-depleted environments and at elevated temperatures. Our results emphasize the widespread implications of declining host-associated microbial diversity from host cellular physiology to ecosystem nutrient cycling. These demonstrated effects of declining microbiome diversity are likely to be amplified in ecosystems experiencing multiple stressors caused by anthropogenic activities.

**IMPORTANCE:**

As evidence is emerging of the key roles that host-associated microbiomes often play in regulating the physiology, fitness, and ecology of their eukaryotic hosts, human activities are causing declines in biological diversity, including within the microbial world. Here, we use a multifactorial manipulative experiment to test the effects of declining diversity within host microbiomes both alone and in tandem with the effects of emerging global changes, including climate warming and shifts in nutrient bioavailability, which are inflicting increasing abiotic stress on host organisms. Using single-celled eukaryotic phytoplankton that harbor an external microbiome as a model system, we demonstrate that diversity within host-associated microbiomes impacts multiple tiers of biological organization, including host physiology, the host population and community ecology, and ecosystem nutrient cycling. Notably, these microbiome diversity-driven effects became magnified in abiotically stressful environments, suggesting that the importance of microbiome diversity may have increased over time during the Anthropocene.

## INTRODUCTION

All eukaryotes harbor microbiomes and these host-associated microbes are often key regulators of host physiology and fitness (1–3). Host-associated microbes can also modulate the behavior and population ecology of their hosts, with cascading implications on the ecological interactions among hosts (4–7). However, as evidence is emerging of the critical roles that microbiomes play, human activities are causing biological diversity declines, including within the microbial world (8–10). Therefore, we tested the effects of declining diversity within host microbiomes both alone and in combination with the effects of other global changes that are inflicting abiotic stress on host organisms, specifically climate warming and shifts in nutrient bioavailability.

We use phytoplankton and their associated bacteria as a model system to address this question because (i) these single-celled eukaryotes harbor external microbiomes that are amenable to experimental manipulation, (ii) phytoplankton serve a critical role in aquatic food webs and the global carbon cycle, and (iii) phytoplankton model systems have played a key role in the development of community ecology theory ever since the proposal of the paradox of the phytoplankton by G. E. Hutchinson (11–14). Using this tractable model system, we aim to elucidate the importance of taxonomic and phenotypic diversity within microbiomes for a broad range of host organisms. Applications of this work within phytoplankton include improvements in the production of phytoplankton-based biofuels and biopharmaceuticals (15). Such model systems could also be applied to organisms less amenable to manipulation, like wild plant and animal host populations in natural ecosystems, or even contribute to clarifying the effects of microbiome diversity on human health, as humans harbor increasingly depauperate microbiomes due to modern diets, use of antimicrobial drugs, and residence in relatively sterile human-built rather than natural environments (10, 16).

Furthermore, it is essential to understand the fundamental regulators of phytoplankton ecology because phytoplankton are responsible for half of global oxygen production (14) and form the base of aquatic food webs (17, 18). Therefore, changes in phytoplankton cellular stoichiometry, population abundances, and community composition could have cascading effects on higher trophic levels and biogeochemical cycles. Phytoplankton harbor bacteria and other microbes within the phycosphere, a microhabitat enriched in sugars and other byproducts of photosynthesis that immediately surround the host cell (19). The concentrated gradient of exudates surrounding the phytoplankton cell attracts a diversity of bacteria from the surrounding environment via chemotaxis (19, 20). Phytoplankton-bacterial interactions within the phycosphere have been found to range from mutualism and commensalism to competition and parasitism (19). Furthermore, phytoplankton-associated bacteria have been found to regulate phytoplankton fitness, population ecology, and even community dynamics (7, 21). Specifically, phytoplankton monocultures have attained greater carrying capacities when grown with, versus without, their microbiome (21, 22). In addition, the presence versus absence of phycosphere bacteria has been shown to mediate ecological interactions between species of phytoplankton hosts, with phycosphere bacteria tending to promote phytoplankton coexistence in pairwise mutual invasibility experiments (7). We now expand upon this work by testing how the diversity of bacteria within the phycosphere affects phytoplankton physiology, fitness, and community dynamics within more complex multi-species communities.

We test the importance of diversity within the host microbiome against temperature and the bioavailability of nutrients in ecosystems because these two abiotic factors are both known to be important regulators of phytoplankton abundance and diversity and are changing due to human activities (23, 24). Climate change-mediated increases in temperatures are expected to decrease phytoplankton diversity, whereas predictions for the effect of temperature increase on phytoplankton abundance are context dependent on the specific ecosystem and taxon of phytoplankton (23, 25–27). Eutrophication, caused by the accumulation of excess phosphorus and nitrogen in aquatic systems from human activities, tends to increase phytoplankton abundance but decrease phytoplankton diversity (28–30). By contrast, nutrient-poor conditions or oligotrophication, caused by efforts to curtail nutrient runoff as well as the introduction of invasive dreissenid mussels, tends to decrease phytoplankton abundance and increase phytoplankton diversity (28, 30–33).

We employed a 3 × 4 × 2 multifactorial design in which phytoplankton communities were exposed to three microbiome diversity treatments, four concentrations of phosphorus, and two temperatures. With this design, we aimed to test how host-microbiome diversity alters phytoplankton stoichiometry, population and community-wide abundance, phytoplankton diversity, and ecosystem-level nutrient cycling. To specifically test the effects of host-associated microbiomes, rather than communities of aquatic microbes that may or may not associate with phytoplankton, we build upon a prior experiment in which phytoplankton hosts were cleaned of microbes and then exposed to microbial communities from aquatic ecosystems to permit recruitment of natural microbiomes. This prior work demonstrated that these phytoplankton microbiomes consist of a subset of taxa recruited from these aquatic ecosystems, are highly host-species specific, and confer fitness benefits to their host (21). By leveraging these experimentally assembled microbiomes in this model system, we now aim to advance our broader understanding of how host-microbiome diversity modulates host health in complex environments.

## MATERIALS AND METHODS

For more details on the experimental methods and statistical analyses, see the supplement. In this study, we used five-species phytoplankton communities by drawing from our prior work to generate axenic (i.e., free of all bacteria) and xenic cultures of *Chlorella sorokiniana*, *Coelastrum microporum, Monoraphidium minutum*, *Scenedesmus acuminatus*, and *Selenastrum capricornutum*. Four of these unicellular green phytoplankton belong to the order Sphaeropleales while *C. sorokiniana* belongs to the order Chlorellales. We chose these fives species of phytoplankton because each is common throughout aquatic habitats of the USA, occurring in 9.3%–55.0% of the ~1,100 lakes surveyed via the US Environmental Protection Agency’s 2007 National Lake Assessment. Furthermore, this species pool includes a range of interaction types, ranging from competition to ecological facilitation (7, 34). Therefore, phytoplankton monocultures of these five species were rendered axenic and used in a microbiome assembly study as described by our prior work (7, 21). In brief, initially, axenic monocultures acquired freshwater bacterial communities when submerged in pond water collected from a long-term experimental pond facility in Pinckney, Michigan, USA (21, 35). For this current study, we obtained phytoplankton microbiomes from monocultures that had assembled microbial communities from Pond 2 and Pond 3 (Fig. S1a). These ponds contained distinct bacterial communities, which resulted in phytoplankton monocultures recruiting distinct microbiomes from each pond (Fig. S1b). These phytoplankton-associated microbiomes were then used in the current study to inoculate axenic phytoplankton communities with three levels of microbiome diversity.

### Experimental design

**Fig 1:**
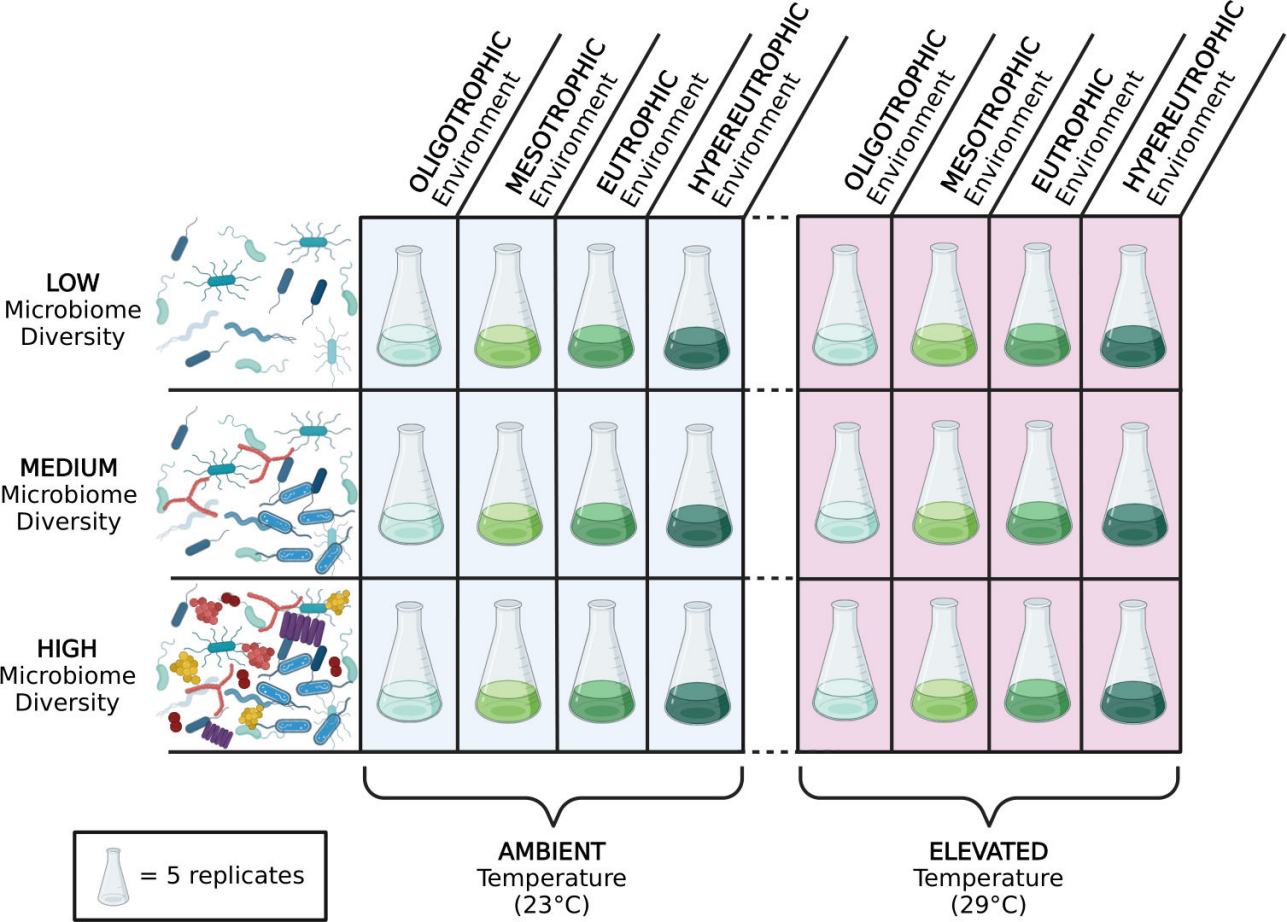
Illustration of experimental design that includes microbiome diversity, phosphorus concentration, and temperature treatments. All 120 flasks were inoculated with the identical five-species community of phytoplankton, with five replicates assigned to each of the 24 treatment combinations. This illustration was created using BioRender.

We carried out a 6-week experiment using a 3 × 4 × 2 multifactorial design to test for the independent and interactive effects of diversity within the host microbiome, lake phosphorus concentration, and water temperature on metrics spanning from host physiology to ecosystem nutrient cycling (Fig. 1). We had five biological replicates per treatment combination for a total of 120 flasks. Each flask contained 100 mL of sterile COMBO plankton growth media (36) at the corresponding phosphorus concentration and was inoculated with each of the five phytoplankton species to create an axenic community with a total cell density of ~12,000 cells/mL.

For detailed methods and an illustration of how we created the microbiome diversity treatments, see the supplemental text and Fig. S2. In brief, for each of the five phytoplankton species described above, we grew an axenic monoculture, a xenic monoculture with a microbiome recruited from Pond 2, and a xenic monoculture with a microbiome recruited from Pond 3 in full-strength COMBO media. These 15 monocultures were used to create phytoplankton-associated bacterial communities as described in our prior work by separating bacteria cells from host cells using 3.0 µm filters (21). To inoculate axenic phytoplankton communities with freshwater bacteria found in association with each of the five host species, we pooled filtrates containing bacterial communities from each phytoplankton monoculture from a respective group (i.e., axenic, Pond 2, and Pond 3). To generate the high microbiome diversity treatment, we used 15 µL of the pooled Pond 2 bacterial communities and 15 µL of the pooled Pond 3 bacterial communities for each of the 40 flasks containing axenic five-species phytoplankton communities. For the medium microbiome diversity treatment, we added 30 µL of the pooled Pond 3 bacterial communities to a second set of 40 flasks. To generate the low microbiome diversity treatment, we added 30 µL of the pooled axenic filtrate (hereinafter referred to as our T_0_ axenic inoculant) to the last set of 40 flasks. Prior to inoculating these five-species phytoplankton communities, we confirmed the axenic status of our stock cultures via visualization of DAPI-stained cultures on a Zeiss Axio Imager 2 fluorescence microscope. We also used 16S rRNA amplicon sequencing (as described in detail below) to characterize bacterial community composition, richness, as well as the taxonomic overlap of each of our three inoculants containing bacterial communities at the time of inoculation. Results from 16S rRNA sequencing of our T_0_ axenic inoculant were consistent with axenic biomass, yielding fewer total reads and similar taxonomic richness compared to control blanks that were processed simultaneously to control for contamination during library preparation and sequencing (see Fig. S3 and 4; Table S1). However, we acknowledge that despite the use of aseptic technique, our axenic flasks became contaminated with a small number of bacterial taxa likely due to repeated sampling and media replenishment. Therefore, we refer to this treatment as “low microbiome diversity” from here onward. These bacteria residing within the low microbiome diversity treatment originated nearly entirely from phycosphere bacterial communities inhabiting the other two tiers of our microbiome diversity treatment. Specifically, 48.98% of the bacterial taxa and 77.23% of the total sequencing reads of bacteria inhabiting the low microbiome diversity flasks by the end of the 6-week study originated from the medium and high microbiome diversity flasks according to Bayesian SourceTracker (37) (Fig. S5; Table S2).

For our phosphorus treatment, we used sterile plankton growth media, COMBO, that we modified to range from oligotrophic to hypereutrophic nutrient conditions (i.e., aquatic trophic state) using the following percentages of the K_2_HPO_4_ stock solution: 0% for oligotrophic media; 1% for mesotrophic; 2% for eutrophic; and 10% for hypereutrophic. Total phosphorus concentrations of our starting media ranged from 5.0 to 310 µg/L, which encompasses the range of total phosphorus documented in over 95% of lakes in the northeastern United States (38).

All 120 flasks were incubated at their corresponding temperature treatment on shaker tables set to 80 RPM and under 81 μE lighting with a 16:8 hour light-dark cycle with the spatial location of flasks randomized by microbiome diversity and phosphorus treatments. To generate the elevated temperature treatment, heat mats were set to a constant temperature, resulting in average daytime temperatures of 28.8 ± 0.03°C SE throughout the duration of the study, which contrasted with the ambient treatment maintained at 23.0 ± 0.02°C SE.

### Bacterial community dynamics

With recent advances in flow cytometry, we can now measure phenotypic diversity metrics of taxonomically rich communities of bacteria. Critically, these phenotypic diversity metrics correlate with taxonomic diversity as measured via amplicon sequencing but require only small sample volumes (39). To obtain an independent measure of bacterial diversity for each of our five biological replicates per treatment combination, we determined bacterial phenotypic diversity at the end of the 6-week experiment for all 120 flasks using a BD FACSCanto II system. We gated the bacterial populations using the FITC-A and the PerCP-Cy5.5 fluorescence channels. While a percentage of bacterial cells that remained adhered to a phytoplankton cell would have been excluded from this bacterial gate, we expect this occurrence would have been consistent across experimental treatments and would therefore not have caused systematic bias in our estimates of bacterial phenotypic diversity. Furthermore, we verified that there was minimal cell aggregation within the bacterial gate by plotting area versus height dot plots using the FITC-A and FITC-H channels. Lastly, we quantified bacterial phenotypic diversity using the Hill number 2 (^2^D, Inverse Simpson index) calculated with the Phenoflow analysis package in R (39).

To further characterize bacterial community composition at the end of the 6-week experiment, we also carried out taxonomic sequencing for each of our 24 treatment combinations. We pooled our five biological replicates per treatment combination due to a limited volume per flask. We collected biomass from all five biological replicates per combination onto a single 0.22 µm nitrocellulose filter and extracted DNA from filters using a Qiagen DNeasy Blood and Tissue Kit. This filter size ensured that we captured all bacterial taxa, including those most closely associated with phytoplankton, which may be embedded in the mucosal matrix surrounding phytoplankton cells. We used the same filtering and extraction methods for our T_0_ samples of the Pond 2, Pond 3, and axenic groups used to inoculate all flasks. The V4 region of the 16S rRNA gene was amplified using 515f/806r primers and sequenced on a 2 × 150 Illumina MiSeq run at the UCSD Institute for Genomic Medicine (40). To facilitate the interpretation of low biomass samples, we also included controls during library preparation and sequencing, including four no-template-control blanks and a four-sample dilution series of a ZymoBiomics Microbial Community Standard. We processed reads using QIIME2, DADA2, and phyloseq (41–43). Merged reads were assigned taxonomy using the SILVA 138 reference database (44, 45). From 24 samples, which represent bacterial communities from each of our unique treatment combinations after 6 weeks, we recovered 141 ASVs at a median of 13,655 bacterial reads after removing phytoplankton chloroplast reads but prior to rarefaction.

### Phytoplankton stoichiometry, morphology and ecology

To measure the initial host cell stoichiometry of axenic stocks, we cleaned cell pellets of COMBO media using serial separation-by-centrifugation with sterile NaCl saline solution. Each washed cell pellet was then collected onto a pre-weighed, pre-combusted Whatman GF/F filter, filters were dried for 48 hours at 60°C, weighed to determine phytoplankton biomass, and stored for isotopic analysis. We measured final host cell stoichiometry from each flask after 6 weeks using this same method with the exception that an additional sonication step was added to dislodge bacteria from the mucilage of the phycosphere. While our sonication and serial separation-by-centrifugation method should have removed most bacterial cells, we acknowledge that our phytoplankton biomass may have retained trace amounts of bacterial cells. Phytoplankton biomass was then scraped off of filters and ground using a mortar and pestle. We packed 3 mg of powdered phytoplankton biomass into 3.5 × 5 mm tin capsules and measured C:N, δ^15^N, and δ^13^C using a PDZ Europa ANCA-GSL elemental analyzer interfaced to a PDZ Europa 20–20 isotopic ratio mass spectrometer at the University of California Davis Stable Isotope Facility.

To determine how cell morphology varied by treatment, including cell area, diameter, height, and perimeter of each phytoplankton species, we randomly selected two flasks per treatment combination for analysis using an Amnis ImageStream^X^ Mk II Imaging Flow Cytometer (Cytek Biosciences, Fremont, California, USA). Cell morphology data were then processed using random forest and convolution neural networks to classify data by phytoplankton species using Amnis AI Software.

To determine how phytoplankton cell density and species composition varied by treatment at the end of the 6-week study, we counted either 400 cells or four hemocytometer grids (3.6 µL), whichever came first, of each species per flask (46). Finally, we measured phytoplankton biomass after 6-weeks using the methods described above for stoichiometry.

### Ecosystem nutrient cycling

We determined the concentrations of total dissolved nitrogen and phosphorus remaining in the spent growth media for each of the 120 flasks at the end of our 6-week study by processing samples at the Marine Chemistry Lab of the University of Washington’s School of Oceanography using the Valderrama 1981 protocol (47).

### Statistical analysis

To test whether bacterial phenotypic diversity varied across microbiome diversity treatments, we used *a priori* ordered predictions to calculate a directional ANOVA using Spearman’s rank correlations (48). We then used 16S amplicon data to test the relationship of bacterial community composition and phylogenetic membership with each experimental treatment using the quantitative Jaccard and weighted UniFrac distance matrices, respectively, with a distance-based redundancy analysis and permutational analysis of variance. We then used a multiple linear regression to determine whether bacterial alpha diversity was predicted by our three treatments: microbiome diversity, phosphorus treatment, and temperature treatment. We also used three-way ANOVA and Tukey’s HSD post hoc tests to evaluate the independent and interactive effects of our three treatments on each of the following dependent variables: δ^15^N and δ^13^C of phytoplankton biomass, cell abundance of each of the five phytoplankton species, Shannon diversity of the phytoplankton community, and total dissolved nitrogen and total dissolved phosphorus of the media. To measure the effects of the three treatments on phytoplankton cell morphology, we used a multiple linear regression and multivariate analysis of variance (MANOVA) to model the collective outcome of mean area, diameter, height, and perimeter of the cell as predicted by the three treatments and phytoplankton species as fixed effects with interactions between all fixed effects. We also ran separate MANOVAs for each of the five phytoplankton species with fixed and interaction effects for the three treatments. For more information regarding the R packages and functions used to complete statistical analyses, consult the supplemental material and methods text.

To conceptually diagram causal relationships between treatments, outcomes, and co-factors measured within this study, we composed a directed acyclic graph (DAG). We then evaluated causal inference given our experimental design following the four assumptions outlined by Kimmel et al.: excludability, no interference, no multiple versions of treatments, and no noncompliance (49). In brief, our experimental design meets these assumptions by (i) satisfying excludability, whereby the outcomes of our treatments are the result of the factorial interaction of microbiome diversity, phosphorus, and temperature and not a result of another unintended outcome caused by the application of our treatments (see DAG in Fig. S6). (ii) Our experimental design has no interference between experimental flasks (units), meaning the treatment of one flask does not affect the outcome of another independent flask over the course of this 6-week study. (iii) We acknowledge that our factorial design can lead to outcomes conditional on multiple treatment versions; however, we define and carefully conclude at the level of treatment combinations. Lastly (iv) our experimental design satisfies the no noncompliance assumption, through validations with T_0_ and T_F_ measurements that the microbiome diversity, phosphorus, and temperature treatments had their intended effects. We can therefore infer causality for these three main treatments on each of our measurements, but also note the relevant covariates and potential indirect pathways that could be causing these effects in our DAG.

## RESULTS

### Microbiome diversity

**Fig 2:**
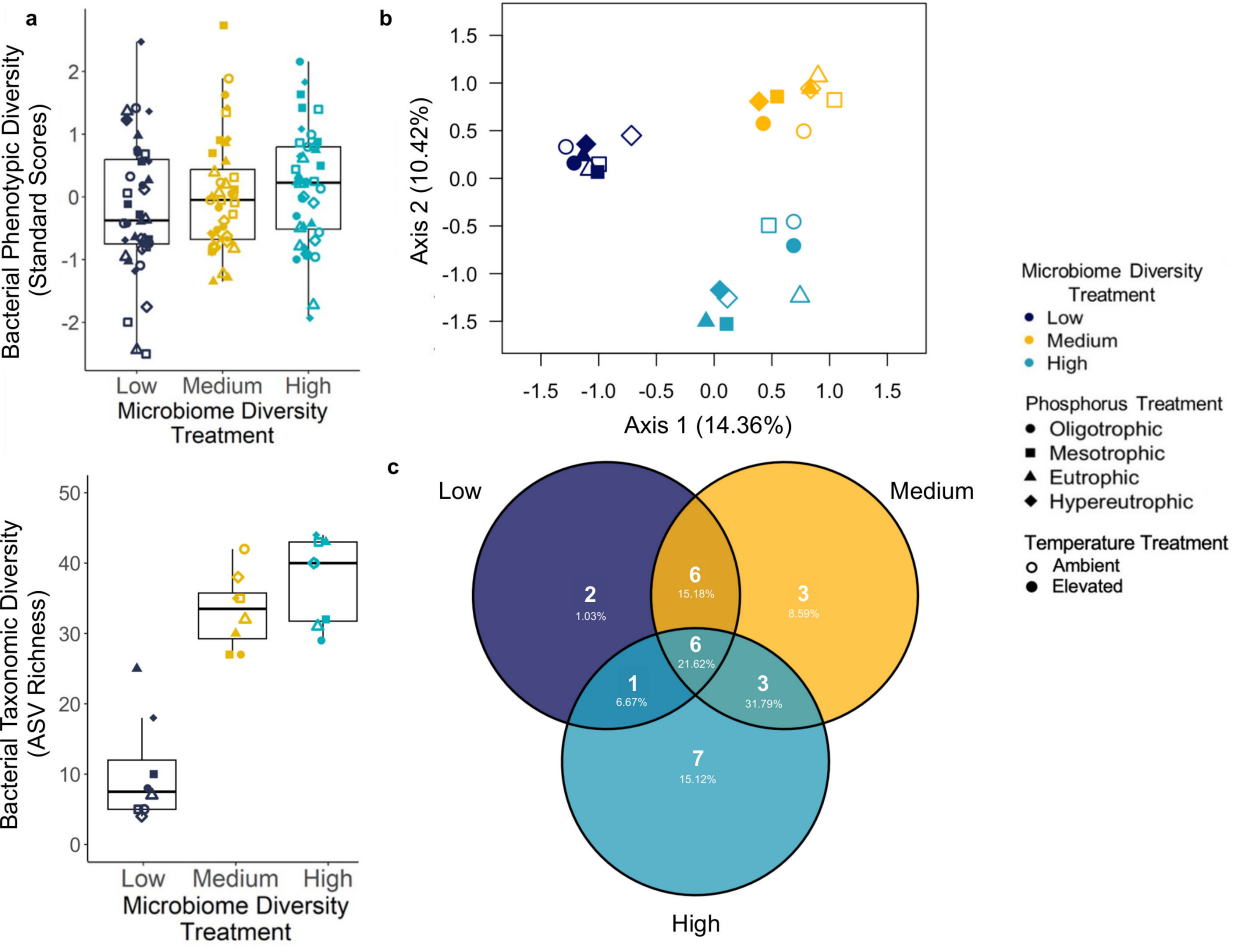
**(a**) The microbiome diversity treatment corresponded with bacterial phenotypic diversity, as measured via flow cytometry for each flask at the end of the 6-week study (directional analysis of variance incorporating *a priori* ordered prediction, *P* < 0.05). Diversity was calculated with an Inverse Simpson’s Index and standardized by a batch of flow cytometry samples, where one biological replicate per treatment combination was run in each batch. Furthermore, bacterial taxonomic diversity also differed among our microbiome diversity treatments (*F*_2,17_=61.69, *P* < 0.0001). Bacterial taxonomic richness was calculated using non-rarefied amplicon sequence data, see Fig. S3 for rarefied results. Replicate flasks assigned the same treatment combination were combined to obtain sufficient volume for amplicon sequencing resulting in a lower sample size for taxonomic analysis. (**b**) The microbiome diversity treatment was the dominant driver of microbiome composition (ANOVA on quantitative Jaccard distance—microbiome diversity: *F*_2,17_=3.73, *P* < 0.0001; phosphorus: *F*_3,17_=1.59, *P* < 0.01; temperature: *F*_1,17_=1.29, *P* > 0.05). (**c**) Despite distinct compositional differences among microbiome diversity treatments, when examining common ASVs only (>1% in proportional abundance within a treatment), 57% of ASVs were shared with at least one other microbiome diversity treatment. In addition, the percent of reads shared among each microbiome diversity is described below the number of ASVs, with the greatest number of shared reads between the medium and high microbiome diversity levels.

While our microbiome diversity treatments were designed to incorporate all microbes smaller than 3.0 µm in size, including both bacteria and fungi, we evaluate the preservation of our diversity treatment levels by focusing on phenotypic and taxonomic diversity of bacteria, rather than fungi, due to the more robust methods presently available for bacteria. Through the end of our 6-week study, the three levels within our microbiome diversity treatment retained significant differences in bacterial diversity, with the directionality as expected, based on two independent experimental techniques. First, bacterial phenotypic diversity, as measured by flow cytometry, demonstrated the lowest diversity within the low microbiome diversity treatment and the highest diversity within the high treatment (Fig. 2a; directional analysis of variance, *P* < 0.05). Second, marker gene sequencing data further showed this treatment effect in bacterial taxonomic diversity, with low, medium, and high microbiome richness levels of the treatment containing: µ = 9.88 ASVs ±2.61 SE; 33.12 ASVs ± 1.83; and 37.38 ASVs ± 2.12, respectively (*F*_2,17_=61.69, *P* < 0.0001; Fig. 2a; Fig. S7). Furthermore, samples within each level of the diversity treatment varied in taxonomic composition, showing that the treatment tested the effects of diversity rather than any specific taxonomic community. Specifically, only 19.75% of the 81 ASVs found in the high-diversity treatment were observed across all high-diversity amplicon sequencing samples, whereas the remaining 80.25% of taxa were found in some but not all of these samples. Similarly, only 20.25% of the 73 ASVs found in the medium diversity treatment were found in each medium diversity sample, while none of the 49 ASVs found in the low diversity treatment were found across all low diversity samples.

Furthermore, the microbiome diversity treatment was the strongest predictor of bacterial community composition among phytoplankton cultures (Fig. 2b; db-RDA using a quantitative Jaccard distance: *F*_2,17_=3.73, *P* < 0.0001; weighted UniFrac distance: *F*_2,17_=4.76, *P* < 0.0001, *n* = 8 per microbiome diversity treatment level). The phosphorus treatment was also a significant predictor of bacterial community composition (Fig. 2b; quantitative Jaccard: *F*_3,17_=1.59, *P* < 0.01; weighted UniFrac: *F*_3,17_ =2.09, *P* < 0.05, *n* = 6 per phosphorus treatment level). This was particularly evident within the medium and high microbiome diversity levels, where the effect of the phosphorus treatment on bacterial community composition reflected a gradient from oligotrophic to hypereutrophic nutrient conditions (quantitative Jaccard: *F*_3,10_=2.75, *P* < 0.01; weighted UniFrac: *F*_3,10_=4.45, *P* < 0.001; Fig. S8). However, by the end of 6-weeks, the temperature treatment had no significant effect on phytoplankton-associated bacterial community composition (quantitative Jaccard: *F*_1,17_=1.29, *P* > 0.05; weighted UniFrac: *F*_1,17_=1.62, *P* > 0.05, *n* = 12 per temperature treatment level). In addition to the effects of our three factorial treatments, differences in bacterial community composition and richness may also be mediated by host factors such as biomass and host diversity (see causal pathways in Fig. S6). Lastly, despite significant divergence across levels of the microbiome diversity treatment, there was a core community of ASVs that were shared among at least two of the three levels of the microbiome diversity treatment (57.14% of all common ASVs, where common was defined as those that comprised at least 1% of the community; Fig. 2c). A summary of bacterial community composition and diversity at the beginning of the study, as well as composition and diversity at the end of the 6-week study across the diversity, phosphorus, and temperature treatments is provided in Table S3 and as heat maps in Fig. S9 and S10.

### Phytoplankton cell stoichiometry and morphology

**Fig 3:**
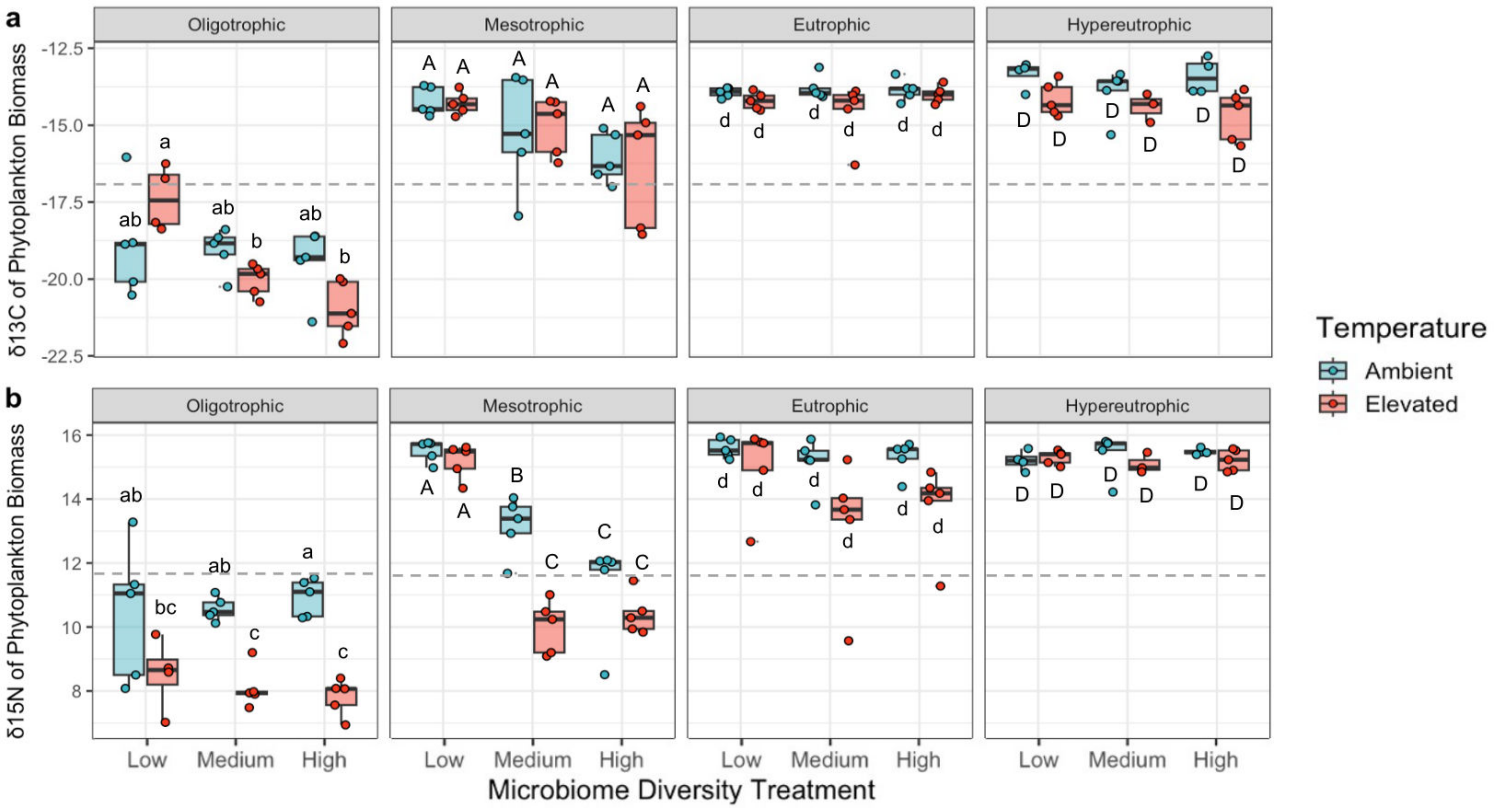
Trophic state, as assigned by our phosphorus treatment, was the dominant driver of (a) mean δ^13^C and (b) δ^15^N of phytoplankton biomass. Microbiome diversity and temperature were significant, but secondary, drivers of phytoplankton δ^13^C and δ^15^N. Biomass was sampled at the end of the six-week experiment where five-species phytoplankton communities underwent a combination of temperature, microbiome diversity, and phosphorus treatments. ANOVA of δ^13^C—microbiome diversity: *F*_2,91_=12.0, *P* < 0.0001, phosphorus: *F*_3,91_=213.3, *P* < 0.0001, temperature: *F*_1,91_=4.3, *P* = 0.042; ANOVA of δ^15^N—microbiome diversity: *F*_2,91_=25.8, *P* < 0.0001, phosphorus: *F*_3,91_=208.8, *P* < 0.0001, temperature: *F*_1,91_=54.6, *P* < 0.0001. See Tables S2 and S3 for ANOVA tables. Dashed lines show the mean values of each metric for the axenic five-species phytoplankton communities that were grown in full-strength COMBO media and used to set up the experiment. Pairwise comparisons were made within each trophic state and statistically similar groups, as determined by four independent Tukey’s HSD tests (i.e., one for each trophic state), are denoted by letters.

The phosphorus, microbiome diversity, and temperature treatments each significantly influenced phytoplankton δ^13^C and δ^15^N. Both isotopic ratios increased markedly with phosphorus concentration (δ^13^C: *F*_3,91_=213.3, *P* < 0.001, Fig. 3a; δ^15^N: *F*_3,91_=208.8, *P* < 0.001, Fig. 3b; Tables S4 and S5). However, within a trophic state, particularly the nutrient-limited oligotrophic and mesotrophic environments, there was a notable decrease of δ^13^C and δ^15^N with increasing microbiome diversity (δ^13^C: microbiome diversity *F*_2,91_=11.96, *P* < 0.001, microbiome diversity × phosphorus *F*_6,91_=3.61, *P* < 0.01; δ^15^N: microbiome diversity *F*_2,91_=25.8, *P* < 0.001, microbiome diversity × phosphorus *F*_6,91_=13.3, *P* < 0.001). While shifts in phytoplankton community composition alone would alter δ^13^C and δ^15^N due to interspecific variation in the isotopic signatures of phytoplankton, shifts in composition alone did not explain these patterns. We demonstrate this by calculating expected isotopic signatures of community-level biomass collected at the week 6 timepoint by combining our data on isotopic signatures of each host species as an axenic monoculture and the proportions of each phytoplankton species in each community at this same timepoint. In oligotrophic and mesotrophic environments, we found that our calculated estimates based on phytoplankton community composition were not significantly different between the low and high microbiome treatments (δ^13^C: low diversity µ = −17.3 ± .057 SE, high diversity µ = −17.5 ± .11, paired ANOVA: *F*_1,37_=4.05, *P* > 0.05; δ^15^N: low diversity µ = 13.2 ± .19, high diversity µ = 13.3 ± .10, paired ANOVA: *F*_1,37_=0.19, *P* > 0.05), whereas our measured results on the biomass obtained in the study were indeed significantly different (δ^13^C: low diversity µ = −16.1 ± .53, high diversity µ = −18.0 ± .53, paired ANOVA: *F*_1,37_=23.92, *P* < 0.01; δ^15^N: low diversity µ = 12.6 ± .74, high diversity µ = 10.2 ± .36, paired ANOVA: *F*_1,37_=14.07, *P* < 0.01). Lastly, phytoplankton isotopic ratios were also significantly greater in the ambient relative to the elevated temperature condition, although this effect of temperature for δ^15^N was context dependent on the microbiome treatments in nutrient-limited environments (δ^13^C: *F*_1,91_=4.3, *P* < 0.05; δ^15^N: *F*_1,91_=54.6, *P* < 0.001; Table S4 and S5). This context dependency is further illustrated in our causal DAG, whereby bacterial microbiome diversity may mediate this relationship between our temperature treatment and phytoplankton δ^15^N (Fig. S6).

In addition, each treatment influenced phytoplankton cell morphology, collectively measured as cell area, diameter, height, and perimeter (MANOVA phosphorus: *F* = 10.26, *P* < 0.001; microbiome diversity: *F* = 2.15, *P* < 0.05; temperature: *F* = 4.71, *P* < 0.001; Fig. S11; Table S6). The phosphorus treatment was the strongest predictor of cell morphology. Under oligotrophic conditions, we found larger cell sizes of *C. sorokiniana*, *M. minutum*, and *S. acuminatus* compared to hypereutrophic conditions, whereas we found the opposite trend of larger cell size under hypereutrophic conditions for *C. microporum*. As phytoplankton species was a significant main effect in our MANOVA, we also report MANOVAs for each phytoplankton species independently (see Table S7 to S11).

### Phytoplankton community dynamics

**Fig 4:**
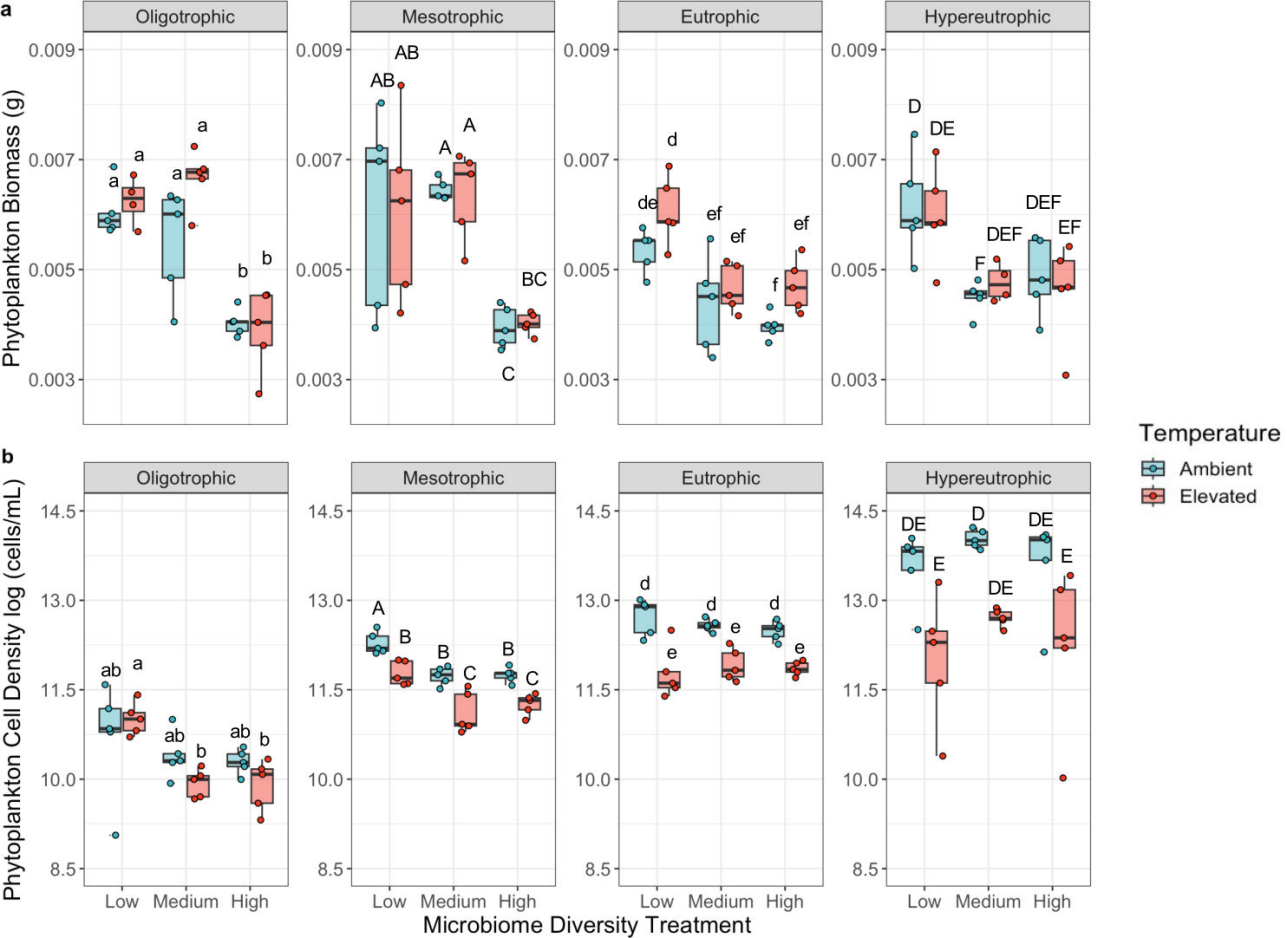
**(a**) Microbiome diversity was the dominant driver of phytoplankton biomass, as measured at the end of the 6-week study, particularly in more phosphorus-limited environments. ANOVA—microbiome diversity: *F*_2,94_=52.0, *P* < 0.001, phosphorus: *F*_3,94_=3.9, *P* = 0.012, phosphorus × microbiome diversity: *P* < 0.0001 (see Table S12 for ANOVA table). (**b**) By contrast, the trophic state was the dominant driver of phytoplankton cell density among the five-species phytoplankton communities. Within each trophic state, microbiome diversity also influenced phytoplankton cell density, with divergent patterns as phosphorous concentrations increased. ANOVA—microbiome diversity: *F*_2,96_=3.7, *P* = 0.030, phosphorus: *F*_3,96_=144.1, *P* < 0.0001, temperature: *F*_1,96_=59.6, *P* < 0.0001 (see Table S13 for ANOVA table). Pairwise comparisons were made within each trophic state and statistically similar groups, as determined by four independent Tukey’s HSD tests (i.e., one for each trophic state), are denoted by letters.

Among the three experimental treatments tested, the microbiome diversity treatment had the greatest effect on total phytoplankton biomass with bacterial diversity inversely correlated with total biomass, while our phosphorus treatment was a significant but secondary factor (Fig. 4a; microbiome diversity: *F*_2,94_=51.98, *P* < 0.001; phosphorus: *F*_3,94_=3.86, *P* < 0.05). Specifically, low microbiome diversity yielded over 40% greater biomass compared to high microbiome diversity. By contrast, the phosphorus treatment was the stronger predictor of phytoplankton cell density, while microbiome diversity was a secondary driver and inversely correlated with cell density (microbiome diversity: *F*_3,96_=3.65, *P* < 0.05; phosphorus: *F*_3,96_=144.1, *P* < 0.001). Specifically, hypereutrophic conditions resulted in phytoplankton cell densities that were on average 17.6 times greater than densities found in oligotrophic conditions. The effects of microbiome diversity in suppressing total biomass and cell density were particularly notable in lower nutrient environments (total biomass: microbiome diversity × phosphorus *F*_6,94_=7.13, *P* < 0.001; cell density: microbiome diversity × phosphorus *F*_6,96_=3.66, *P* < 0.01). For example, microbiome diversity caused a decline in total cell density in oligotrophic conditions; however, when nutrient levels were high, cell density was greatest with intermediate microbiome diversity (Fig. 4b). In addition, ambient temperature conditions yielded phytoplankton cell densities that were on average 2.7 times greater than densities found in the elevated temperature treatment but had no significant effect on total biomass (cell density: *F*_1,96_=59.6, *P* < 0.001; total biomass: *F*_1,94_=2.46, *P* = 0.12; Table S12 and S13).

**Fig 5:**
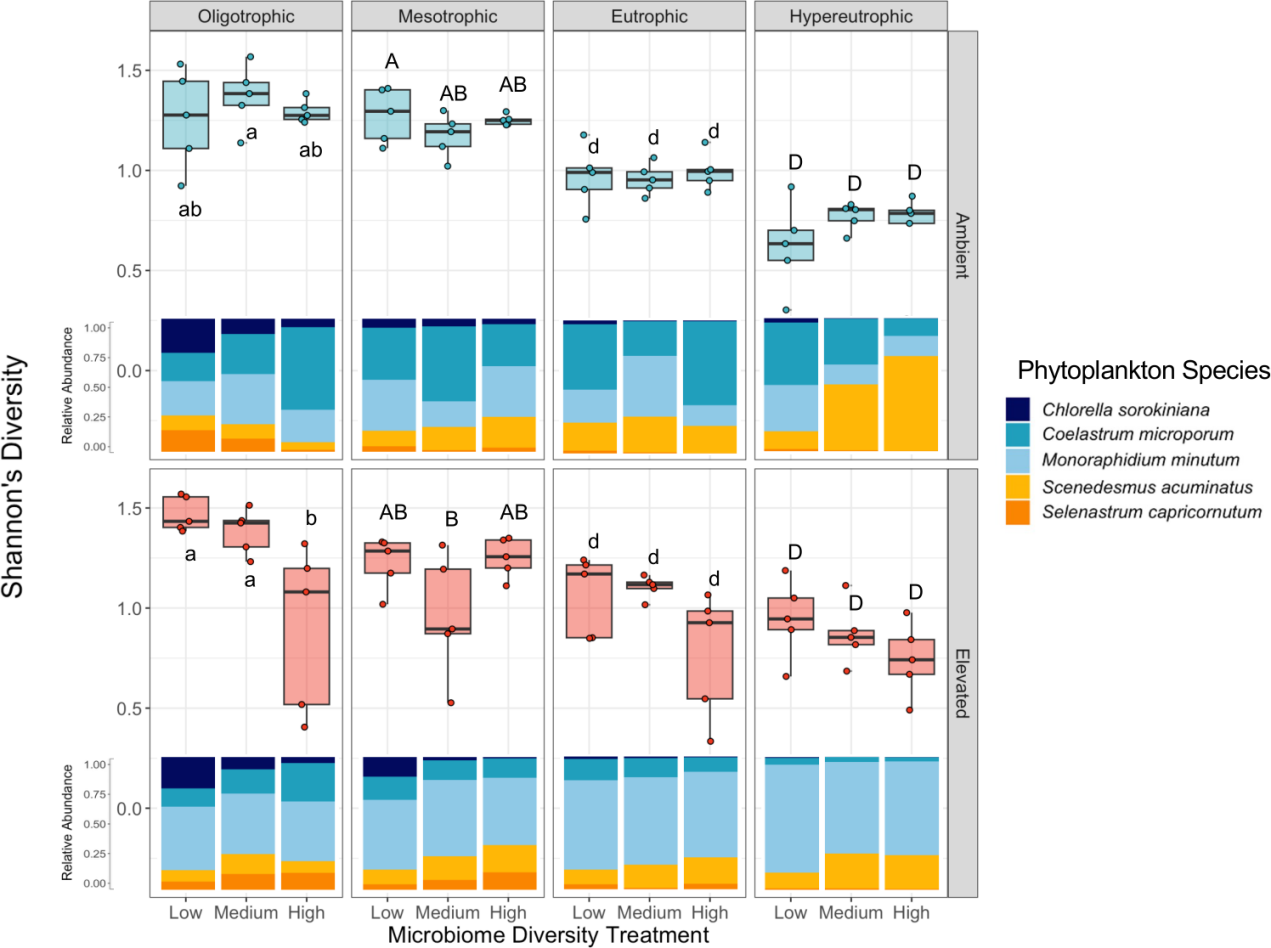
Trophic state, as defined by phosphorus concentration, was the main driver of phytoplankton diversity, whereas microbiome diversity was a significant but secondary driver of phytoplankton diversity (ANOVA—microbiome diversity: *F*_2,96_=4.2, *P* = 0.018, phosphorus: *F*_3,96_=44.6, *P* < 0.0001, temperature: *F*_1,96_=0.04, *P* = 0.85). See Table S14 for the ANOVA table. The mean relative abundance of each of the five phytoplankton species is represented as a stacked bar chart for each treatment combination. Pairwise comparisons were made within each trophic state and statistically similar groups, as determined by four independent Tukey’s HSD tests (i.e., one for each trophic state), are denoted by letters.

Furthermore, phytoplankton diversity was affected by each of the three treatments (Fig. S6). Phosphorus concentration, as mediated by our phosphorus treatment, was negatively correlated with Shannon diversity of the phytoplankton community (*F*_3,96_=44.6, *P* < 0.001; Table S14). For example, all five species of phytoplankton tended to persist under oligotrophic conditions, whereas only three tended to persist under hypereutrophic conditions with the near extinction of *C. sorokiniana* and *S. capricornutum* in higher nutrient conditions (*C. sorokiniana: F*_3,96_=18.8, *P* < 0.001; *S. capricornutum: F*_3,96_=2.11, *P* = 0.10). Within a trophic state, microbiome diversity was negatively correlated with phytoplankton community diversity with this trend particularly notable in lower nutrient environments and at elevated temperatures (microbiome diversity: *F*_2,96_=4.2, *P* = 0.012; microbiome diversity × phosphorus: *F*_6,96_=3.0, *P* = 0.011; microbiome diversity × temperature: *F*_2,96_=5.9, *P* < 0.01). In particular, high microbiome diversity corresponded with a reduced occurrence of *C. sorokiniana*, *C. microporum*, and *S. capricornutum* (Fig. 5; each *P*-value < 0.05; see Table S15 to S19 for statistical results per species).

### Nutrient cycling

**Fig 6:**
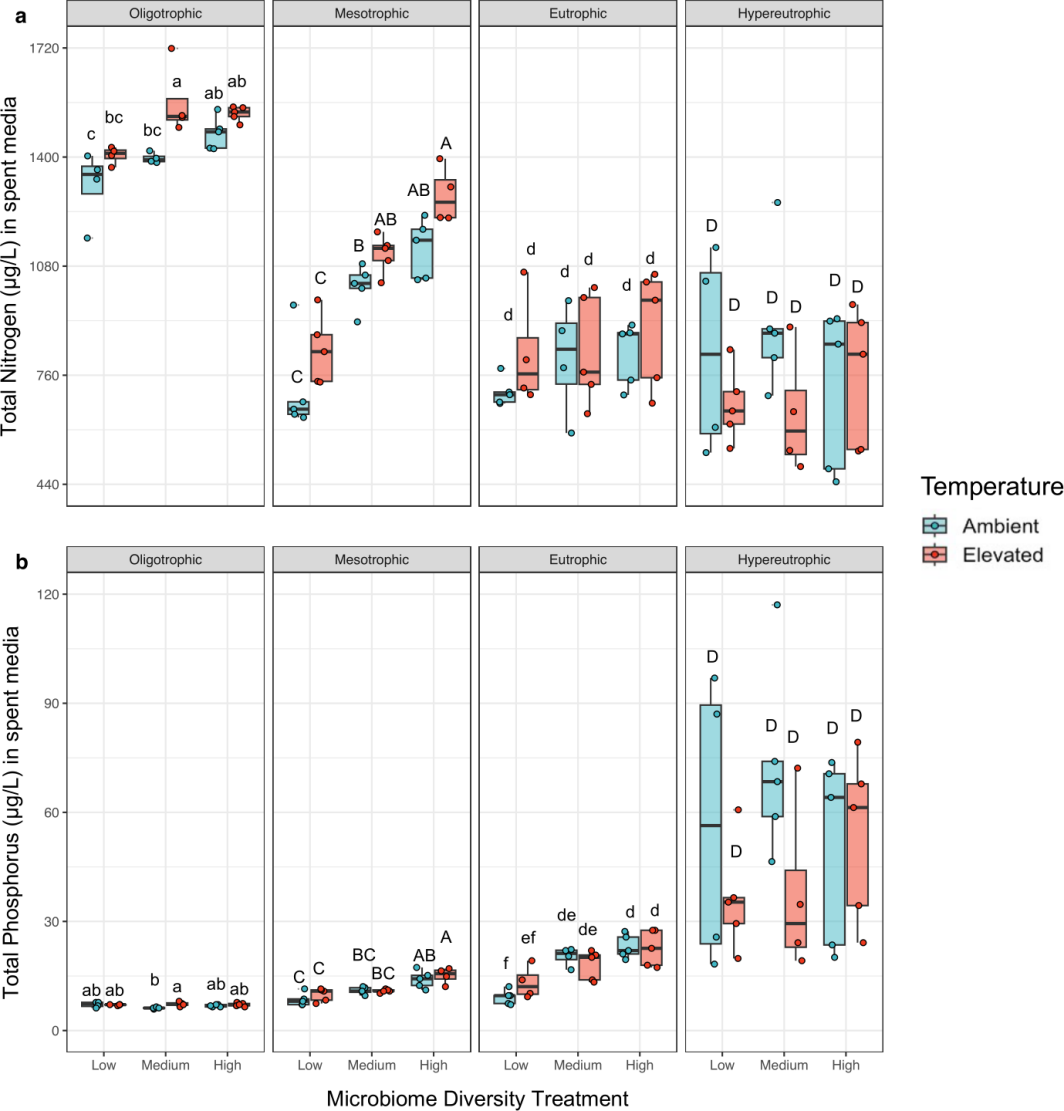
Trophic state, as determined by phosphorus concentration, was the dominant driver of (a) total nitrogen and (b) total phosphorus in spent media, while microbiome diversity was a significant but secondary driver of total N and P concentrations at the end of the 6-week study. Total N and P concentrations were measured of the supernatant from cultures that were pelleted to remove phytoplankton and bacterial cells. ANOVA total nitrogen—microbiome diversity: *F*_2,87_=14.8, *P* < 0.0001, phosphorus: *F*_3,87_=132.9, *P* < 0.0001; temperature: *F*_1,87_=2.2, *P* = 0.13; ANOVA total phosphorus—microbiome diversity: *F*_2,87_=12.45, *P* < 0.0001, phosphorus: *F*_3,87_=173.25, *P* < 0.0001, temperature: *F*_1,87_=0.12, *P* = 0.73. See Table S20 and S21 for ANOVA tables and Fig. S12 to see the comparisons for total phosphorous in an oligotrophic environment. Pairwise comparisons were made within each trophic state and statistically similar groups, as determined by four independent Tukey’s HSD tests (i.e., one for each trophic state), are denoted by letters.

At the end of this 6-week study, the phosphorus treatment contributed to differences in total dissolved nitrogen and phosphorus with the oligotrophic treatment resulting in N concentrations nearly two times greater than concentrations found in the hypereutrophic treatment (Fig. 6; Nitrogen: *F*_3,87_=132.9, *P* < 0.0001, Table S20; Phosphorus: *F*_3,87_=173.3, *P* < 0.0001, Table S21). Total dissolved phosphorus concentrations were positively correlated with the phosphorus treatment, which was expected because our starting hypereutrophic condition contained 32 times greater phosphorus than our oligotrophic condition. However, throughout the experiment, the difference in phosphorus concentration among trophic states declined with the final hypereutrophic condition containing only 7.5× greater phosphorus than the oligotrophic condition.

More strikingly, within a trophic state, microbiome diversity corresponded strongly with dissolved nutrient concentrations (Fig. 6; nitrogen: *F*_2,87_ = 14.8, *P* < 0.0001 and phosphorus: *F*_2,87_ = 12.45, *P* < 0.0001). Specifically, high microbiome diversity corresponded with a 20% and 33% increase in the total dissolved nitrogen and phosphorus concentrations when compared to the low diversity treatment. In addition to a potential direct effect of microbiome diversity on nutrient concentrations, these results could be mediated via indirect effects of microbiome diversity on phytoplankton biomass (see causal pathways in Fig. S6).

## DISCUSSION

We show that diversity within host-associated microbiomes can have significant implications for multiple tiers of biological organization, including phytoplankton physiology, phytoplankton population and community ecology, and ecosystem nutrient cycling. Particularly, we find that these effects caused by microbiome diversity can become magnified in abiotically stressful environments.

### Microbiome diversity effects on host physiology

We show that microbiome diversity can affect host cell stoichiometry and morphology. Specifically, greater microbiome diversity corresponded with reduced cellular stress among phytoplankton, where phytoplankton co-cultured with high diversity microbiomes show more negative δ^13^C and δ^15^N. As phototrophs preferentially assimilate lighter isotopes, more negative or lighter isotopic signatures are indicative of increased isotopic fractionation due to increased resource availability (50–53). A more negative δ^13^C of phytoplankton biomass is often indicative of greater CO_2_ availability (52), which could have been a consequence of higher respiration rates that are known to occur among more diverse bacterial communities (54). The more negative δ^15^N of phytoplankton biomass that was prevalent among our high microbiome diversity treatment may have been the result of increased N availability caused by bacterial N_2_ fixation and/or enhanced N-cycling. Even though different species of phytoplankton vary in their isotopic signatures, shifts in phytoplankton community composition alone could not explain these isotopic patterns; instead, microbiome diversity appeared to alter isotopic signatures at the intraspecific level of phytoplankton. These effects of microbiome diversity were most evident in abiotically stressful environments, particularly our elevated temperature treatment paired with nutrient stress in oligotrophic and mesotrophic conditions. Due to the well-established positive relationship between diversity and ecosystem function, we had expected that microbiome diversity would promote nutrient cycling and therefore alleviate host nutrient stress. Our results corroborate this phenomenon and suggest that greater microbiome diversity did increase total N and P in the environment, as shown in Fig. 6. However, this increase in total N and P could also be the consequence of lower phytoplankton abundance observed in the high microbiome diversity treatment. Furthermore, considering that we also observed reduced N stress among phytoplankton hosts in the high diversity treatment, particularly in mesotrophic environments, these results could indicate that hosts were most limited by nutrients other than N and P, or that diverse bacterial communities were capable of more thorough resource extraction, leading to greater competition with phytoplankton. Overall, these results add to the growing evidence that microbiomes may have particularly important implications for host health when hosts are under stress, similar to studies of plants and insects experiencing drought stress (55, 56).

In addition, microbiome diversity had widespread implications for host cell morphology. We hypothesized this link between diversity and morphology due to the roles of bacteria and host cell morphology in mediating nutrient acquisition. For example, the surface-area-to-volume ratio can regulate the acquisition of nutrients by a host cell and be an indicator of host nutrient stress (57). Smaller cells are advantageous in nutrient-depleted environments by facilitating increased nutrient diffusion per unit of cell volume (57). We found this expected pattern of reduced cell sizes in nutrient-depleted environments for one of the five species tested, *C. microporum*. However, overall, the directionality of the observed effects of microbiome diversity on host cell morphology were host species-specific and the magnitude of these effects tended to be weaker than the effects of our phosphorus or temperature treatments. Nonetheless, our finding of significant effects of diversity on morphology for four of the five host species tested shows that the microbiome has important implications for the regulation of host cell morphological plasticity. While other studies have found that host cell morphology can be regulated by the introduction of individual strains of pathogenic bacteria, our results now demonstrate how diversity within a host-microbiome can have similar effects on host cell morphology (58–60).

### Microbiome diversity effects on phytoplankton population and community ecology

We also found that microbiome diversity corresponded with reduced phytoplankton community cell density, biomass, and diversity. As with our findings that the effects of microbiome diversity on host physiology were magnified under nutrient limitation, we found that the negative correlation between microbiome diversity and both phytoplankton diversity and abundance tended to be strongest in the most abiotically stressful conditions (i.e., oligotrophic environments under elevated temperatures). These results illustrate that the effects of microbiome diversity on the ecological outcomes for host communities are dependent upon the context of abiotic stressors. This aligns with studies of plant-soil systems that have found that microbiome networks are often destabilized by abiotic stressors, with microbes most often exacerbating the effects of stress on their host organisms (61, 62). However, studies have also found the converse, where host microbiomes can alleviate stress, and can confer the greatest magnitude of beneficial effects under the most stressful environmental conditions (62, 63).

However, the directionality of these effects of microbiome diversity on phytoplankton community cell density, biomass, and diversity contrast with our expectations as drawn from prior work in this system. Our past work on phytoplankton monocultures indicated that populations maintained with diverse microbiomes as well as single bacterial isolates tended to attain higher carrying capacities compared to axenic phytoplankton, whereas here we find that microbiome diversity suppressed phytoplankton cell densities in five-species communities (21, 22). We had also expected that a higher diversity of bacteria might sustain a more diverse community of host species due to the host-specificity of microbiome assembly in this system (21). Furthermore, we previously found that the presence versus absence of a microbiome most often facilitated pairwise phytoplankton species coexistence and reduced competitive exclusion, whereas here we find microbiome diversity is inversely correlated with phytoplankton community diversity (7). As has been noted in other studies, these results from more complex five-species phytoplankton communities emphasize the potential challenges with extrapolating complex dynamics in diverse systems from experimental designs that use lower levels of diversity than would be found in natural systems (64–66).

Beyond our work with these specific taxa of phytoplankton and bacteria, most studies in aquatic systems have probed for trends between bacterial diversity and phytoplankton productivity and diversity using correlative surveys in natural systems, which are challenging for inferring causation and have yielded varied results (67–70). However, one study of aquatic mesocosms that manipulated nutrients to create a productivity gradient found that bacterial richness was indeed inversely correlated with host species richness, as was found in our study (71). Overall, although several field and lab-based ecological studies have documented significant correlations between phytoplankton primary productivity and diversity and bacterial diversity, the shape of these relationships has varied across studies and may be context dependent on bacterial taxa.

In contrast to these more inconclusive findings in aquatic ecosystems, there is substantial evidence in vertebrates that diversity within host microbiomes tends to promote host health (72–74). Furthermore, in human microbiomes, which have been studied more exhaustively than most natural ecosystems, clear associations have been found between depleted diversity within gut and skin microbiomes and the incidences of diseases and biomarkers of reduced health (75–78). In contrast with this existing literature, there are several potential explanations for why microbiome diversity did not promote host population growth in our study. First, most studies evaluating the role of standing microbiome diversity in promoting host health are testing resilience to a challenge with a specific host-pathogen, whereas we measured host health metrics in a closed system without specifically introducing known pathogens (74, 75, 79). Furthermore, it is conceivable, that compared to axenic conditions, even the microbial communities comprising our low-diversity treatment were sufficiently diverse to facilitate host health and population growth. For example, phytoplankton microbiomes can promote the bioavailability of N through the fixation of atmospheric nitrogen. While certain bacterial taxa known for their capacity to fix N, such as the Azospirillaceae, were restricted to only medium and high diversity treatments, other such taxa, including the Rhizobiales, were common in the low diversity treatment. If sufficient functional benefits are provided by the low diversity treatment, any additional gain of diversity may have increased the likelihood of exposure to host pathogens, resulting in a relative reduction in host population growth. Lastly, our lack of evidence that higher microbiome diversity promotes host population growth could have been due to the tendency for many ecological interaction types to be difficult to detect due to context dependency. This context dependency is particularly true for synergistic interactions, which may be especially common within syntrophic bacterial communities, compared to antagonistic interactions (80, 81).

### Microbiome diversity effects on ecosystem nutrient cycling

We also found that microbiome diversity was positively correlated with the concentration of total dissolved nitrogen and phosphorus in the water column. In combination with our isotopic evidence that high microbiome diversity decreased phytoplankton stress but did not increase phytoplankton biomass or cell density, we can infer that phytoplankton may have been most limited by resources other than CO_2_, N, and P. In addition to the remineralization of N and P, bacteria are known to facilitate the acquisition of iron, cobalamin, and other B vitamins for their phytoplankton hosts (19). Any effects that microbiome diversity may have on nutrient bioavailability could be directly mediated by bacteria, but could also be an indirect consequence of the effects of the microbiome on phytoplankton abundance. An alternative explanation is that while greater microbiome diversity enhances nutrient cycling, these more diverse bacterial communities could also be more capable of greater resource extraction that could result in stronger competition between bacteria and phytoplankton for limiting resources. Regardless of the underlying mechanism, it is clear that the diversity of host-associated microbiomes can have wide-ranging implications on not only host cell physiology but also broader ecological and ecosystem processes.

### Limitations and future directions

We evaluated how microbiome diversity affects multiple tiers of biological organization scaling from the individual host to ecosystem-level nutrient cycling. We showed that microbiome diversity can indeed have significant effects across these scales, particularly in abiotically stressful environments. Examining mechanistically why the effects of microbiome diversity are magnified in abiotically stressful environments, for example through the use of gene expression and metabolomic analyses, is a necessary future direction that could address how host-microbiome interactions shift across stress gradients. Future work could also aim to investigate the effects of microbiome diversity at a higher resolution than the three-tiered treatment used in this study. Ideally, such diversity treatments would be modeled after classic studies in plant community ecology that have demonstrated how plant diversity regulates ecosystem function with the use of substitutive designs that avoid confounding diversity with composition (82–84). However, taking an analogous experimental approach would be exceedingly challenging with the taxonomic richness of natural microbial systems, such as the 141 taxa found in our study, which far exceeds the number of taxa manipulated in these classic studies (i.e., typically a maximum of 24 taxa). Furthermore, considering that the vast majority of bacteria cannot be isolated in pure culture, such experiments would likely need to balance the benefits of a substitutive design with the advantages of replicating the extensive bacterial diversity found in most natural systems. For example, although we have generated a collection of over 350 bacterial isolates from these phytoplankton microbiomes, over 75% are rare members that each comprise <1% of their respective phycosphere community (22). Despite this limitation, it is important to note that taxonomic composition varied substantially within each level of our microbiome treatment. For example, zero of the 49 different ASVs found across our low-diversity samples were ubiquitous across all flasks in the low-diversity treatment. While it is challenging to conclusively infer the absence from genetic sequencing data due to limited sequencing depth and the natural rarity of many bacterial taxa, we have likely overestimated the taxonomic similarity across flasks within each diversity level because we needed to pool biological replicates to obtain sufficient volumes for sequencing. Therefore, although distinct from the controlled designs of classic plant community ecology experiments, we were able to employ two independent methods to quantify bacterial diversity (sequencing and flow cytometry) that, combined, illustrated that our microbiome diversity treatment was effective in testing a wide range of bacterial community compositions within each tier of our microbiome diversity treatment.

A limitation of our study was that we only evaluated microbiome diversity and composition at the beginning and end of our 6-week experiment. Furthermore, while we have well-replicated measures of bacterial diversity at the end of the study via flow cytometry, our replication for our sequencing results was more limited. Still, by pairing these sequencing data with our use of a multifactorial and gradient-based approach for our experimental design, we were still able to draw upon multiple replicates within each of our three main treatments to make robust conclusions about the effects of each treatment on bacterial taxonomic composition. For example, by using a gradient-based approach for our phosphorus treatment, we were able to see a clear ordered shift in bacterial community composition that matched the ordered shift in lake trophic status from oligotrophic through hyper-eutrophic conditions. Such gradient-based experimental approaches are especially useful in illustrating the context-dependent effects of a treatment, such as microbiome diversity, across the wide-ranging environmental conditions that occur in nature (85). Still, future studies that track fine-scale variation in the microbiome, both over time and within treatment combinations, are needed to advance our understanding of how host microbiomes are maintained in environments experiencing multiple abiotic stressors. Lastly, although our low microbiome diversity treatment did not remain fully axenic over the course of the 6-week study, these bacterial taxa largely originated from the same phycosphere communities found in the medium and high microbiome diversity treatments. By introducing a subset of taxa found in higher diversity treatments, each tier of the diversity treatment ultimately harbored a shared core of bacterial taxa originating from the phycosphere. Furthermore, despite this contamination, the three tiers of our microbiome diversity treatment were maintained through the end of the study, as quantified by flow cytometry and amplicon sequencing. More broadly, the implications of this low diversity condition are advantageous relative to a fully axenic condition because this adds to the biological relevance of the study by being more realistic of the variation in microbiome diversity that might be observed under natural conditions.

### Conclusions

Anthropogenic disturbances are increasing in frequency and intensity, with lasting effects on both local and global patterns of biodiversity and in turn, ecosystem function (86). It is therefore essential to unravel the effects of anthropogenic disturbances in isolation and combination with shifting levels of prokaryotic diversity on host health and ecology. Considering the essential role that phytoplankton play as the primary producers of freshwater and marine systems, any effects of prokaryotic diversity on phytoplankton fitness, population ecology, and patterns of diversity and coexistence would likely have cascading implications on other trophic levels (14). Indeed, our results show how microbiome diversity can influence N and P cycling, demonstrating the role that phytoplankton and their associated bacteria often play in regulating biogeochemical and ecosystem nutrient cycling. Therefore, declining diversity within host microbiomes can have wide-ranging implications not only for host physiology and fitness but also for cascading effects on host community dynamics and ecosystem-level nutrient cycling. Our results emphasize that the effects of microbiome diversity on host fitness through ecosystem function may often be magnified in environments experiencing multiple simultaneous stressors, suggesting that the maintenance of prokaryotic diversity may be an important element in the regulation of host and ecosystem health in the Anthropocene.

## Data Availability

The 16S rRNA data sets generated and analyzed during the current study are available in the NCBI Sequence Read Archive (SRA) repository under the BioProject ID PRJNA1032630. All R scripts and csv files are available on this paper’s GitHub repository (https://github.com/jrdickey9/MicrobiomeDiversity).

## SUPPLEMENTAL MATERIAL

Supplemental Material msystems.01462-24-s0001.docx Supplemental text, figures, and tables.

ASM does not own the copyrights to Supplemental Material that may be linked to, or accessed through, an article. The authors have granted ASM a non-exclusive, world-wide license to publish the Supplemental Material files. Please contact the corresponding author directly for reuse.
