# Supplementary material for: Biodiversity within phytoplankton-associated microbiomes regulates host physiology, host community ecology, and nutrient cycling
Source: mSystems. 2025 Jan 28;10(2):e01462-24. doi: 10.1128/msystems.01462-24 (PMC11834400; doi:10.1128/msystems.01462-24)
Supplement: Supplemental Material — Supplemental text, figures, and tables. [file msystems.01462-24-s0001.docx]

SUPPLEMENTAL FILES

**Text S1. Supplemental Materials and Methods:**

We use five-species phytoplankton communities in this study by drawing from our prior work to generate axenic (i.e., free of all bacteria) and xenic versions of: *Chlorella sorokiniana*, *Coelastrum microporum, Monoraphidium minutum*, *Scenedesmus acuminatus*, and *Selenastrum capricornutum*. We previously rendered each monoculture axenic using ultrasonication and fluorescence-activated single-cell sorting onto solid media and confirmed axenic status via fluorescence microscopy, attempted heterotroph isolation on R2A agar, and attempted amplification of the bacterial 16S rRNA gene fragment via colony PCR (1).

Axenic monocultures were then used in a microbiome assembly study as described by Jackrel et al. 2021(2). In brief, initially axenic phytoplankton monocultures acquired freshwater bacterial communities when submerged in aquariums filled with pond water collected from the University of Michigan E. S. George Reserve’s (ESGR) long-term experimental pond facility in Pinckney, MI, USA. Established in 1988, these ponds were naturalized by adding vegetation from a local pond at the ESGR and currently harbor diverse communities of vegetation, invertebrates, and amphibians (2, 3). More specifically, 100 mL of high cell density monocultures for each species of phytoplankton were placed in 4 oz glass jars and sealed with 3.0 μm filters that prevented phytoplankton cells from escaping, but allowed for the free exchange of phytoplankton exudates and bacteria. Separate jars for each species were incubated in water corresponding to each pond for 72 hours, allowing the exudates from each phytoplankton species to attract host-specific bacterial taxa. Xenic cultures from each jar were then used to establish stocks of phytoplankton with naturally recruited microbiomes corresponding to each pond. For the purposes of this current study, we obtained phytoplankton associated microbiomes from host monocultures that had assembled microbial communities from Pond 2 and Pond 3 (see map of experimental pond facility in **Fig. S1a**). These two ponds contained distinct bacterial communities, which resulted in phytoplankton monocultures recruiting distinct phycosphere communities from each pond based on 16S rRNA amplicon sequencing (**Fig. S1b**). These phytoplankton associated microbiomes were used in this study to inoculate our axenic phytoplankton communities with three levels of microbiome diversity. Lastly, while these microbiome communities undoubtedly contained non-bacterial microbes as would be expected for natural ecosystems, we focus all analyses described below on the bacterial fraction of the microbiome.

***Experimental Treatments***

We carried out a 3 x 4 x 2 multifactorial design to test for the independent and interactive effects of diversity within the host microbiome, lake phosphorus concentration, and water temperature on metrics spanning from host physiology to ecosystem nutrient cycling (**Fig. 1**). We had five biological replicates per unique treatment combination for a total of 120 flasks. Each flask contained 100 mL of sterile COMBO plankton growth media (4) at the corresponding phosphorus concentration and were inoculated with each of the five phytoplankton species to create an axenic community with a total cell density of ~ 12,000 cells/mL. We inoculated *C. sorokiniana, C. microporum,* *M. minutum,* and *S. capricornutum* at 2,000 cells/mL, however we inadvertently inoculated *S. acuminatus* at 3,798 cells/mL. These cell densities, determined via hemocytometer, were consistent for each flask and so this deviation for *S. acuminatus* should not bias interpretation of treatment effects. Further, larger phytoplankton cells will likely harbor a greater number of bacteria within their phycosphere (5). Therefore, in addition to reporting cell density of the inoculated community, we also calculated the approximate contribution of each phytoplankton species towards total phycosphere volume. To do this, we estimated cell shape of each species as an ellipsoid and calculated cell volumes using mean length, width and height using imaging flow cytometry data of axenic monocultures grown in hypereutrophic COMBO media at ambient temperature. From this approach, we infer that the total contribution towards phycosphere volume for each species was approximately 3.5% for *C. sorokiniana,* 25.2% for *C. microporum*, 3.5% for *M. minutum*, 61.1% for *S. acuminatus* and 6.7% for *S. capricornutum*.

We created the microbiome diversity treatments as described in **Fig. S2**. For each of the five phytoplankton species, we grew three cultures: an axenic monoculture, a xenic monoculture with a microbiome recruited from Pond 2, and a xenic monoculture with a microbiome recruited from Pond 3, each in full strength COMBO media. These 15 stock monocultures were used to create phytoplankton associated bacterial filtrates from each of these phytoplankton monocultures via sonication and separation-by-centrifugation as described in our earlier work (2). Specifically, to dislodge bacteria from the mucilage of the phytoplankton microbiome, aliquots of each stock culture were gently sonicated on ice at 20% amplitude for 30 seconds, repeated three times with 1-minute rests between sonication, on a Fisherbrand Model 50 Sonic Dismembrator. To separate bacteria from the larger host cells, sonicated stock cultures were then centrifuged at 900 g for 15 minutes. We then filtered the supernatant containing the bacterial cells through a 3.0 μm 25 mm filter to remove any remaining phytoplankton cells. To inoculate axenic phytoplankton communities with freshwater bacteria found in association with each of the five host species, we pooled filtrates containing bacterial communities collected from each phytoplankton monoculture of each respective group (i.e., axenic, Pond 2 and Pond 3), as illustrated in **Fig. S2**.

For our phosphorus treatment, we used sterile plankton growth media, COMBO, that we modified to range from oligotrophic to hypereutrophic nutrient conditions (i.e., aquatic trophic status) by using the following percentages of the NaNO_3_ and K_2_HPO_4_ stock solutions: 10% of the NaNO_3_ and 0% of the K_2_HPO_4_ stock solutions for oligotrophic media; 10% of the NaNO_3_ and 1% of the K_2_HPO_4_ stock solutions for mesotrophic media; 10% of the NaNO_3_ and 2% of the K_2_HPO_4_ stock solutions for eutrophic media; and 10% of the NaNO_3_ and 10% of the K_2_HPO_4_ stock solutions for hypereutrophic media. We confirmed total dissolved phosphorus and nitrogen concentrations in each media type by storing 40 mL aliquots in pre-rinsed bottles at -20°C and sending for analysis at the Marine Chemistry Lab of the University of Washington’s School of Oceanography following the protocol by Valderrama 1981 (6). Specifically, we found that the total phosphorus concentrations of our starting media ranged from 5.0 µg/L for the oligotrophic media to 310 µg/L for the hyper-eutrophic media, which encompasses the range of total phosphorus documented in over 95% of lakes in the northeastern United States (7). A 10% media replenishment was completed once per week with the media type that corresponded to the designated trophic status of each flask.

All flasks were incubated at their corresponding temperature treatment on shaker tables set to 80 RPM and under 81 μE lighting with a 16:8 hour light-dark cycle with the spatial location of flasks randomized by microbiome diversity and phosphorus treatments. Heat mats were used to generate the elevated temperature treatment, resulting in average daytime temperatures of 28.8 ± 0.03°C SE through the duration of the study, which contrasted with the ambient treatment maintained at 23.0 ± 0.02°C SE. Our temperate treatments were monitored every ten minutes throughout the duration of the study, using Onset HOBO pendant temperature/light data loggers that were submerged in flasks of media alongside the flasks used in the study.

To minimize bacterial contamination, all inoculations and handling of flasks throughout the study, including sampling and media replenishments, were completed using aseptic technique in a ThermoScientific 1300 Series A2 biological safety cabinet. Precautions included opening only a single flask at a time and using 70% ethanol to sterilize all surfaces in between handling of each flask.

***Bacterial flow cytometry***

We used flow cytometry to determine bacterial phenotypic diversity at the end of the six-week experiment for all 120 flasks. We sampled 1 mL per flask, which we preserved in 5 μL of 25% glutaraldehyde, snap froze in liquid nitrogen and stored at -80°C until further use. Immediately before processing, samples were diluted to a 1:9 ratio with sterile milliQ water. We added 5 μL of the nucleic acid stain SYBR Green I to each sample, vortexed, and dark incubated for 20 minutes at 37°C. After incubation, we also added 100 μL or 10 μL of 123Count eBeads to each sample to determine total volume analyzed per sample. We processed samples on a BD FACSCanto II system (Becton Dickinson Biosciences, Franklin Lakes, New Jersey, USA) at the Salk Institute of Biological Studies Flow Cytometry Core Facility (La Jolla, California, USA). We analyzed flow cytometry data by gating the bacterial populations using the FITC-A and the PerCP-Cy5.5 fluorescence channels and quantified bacterial phenotypic diversity using the D2 metric calculated with the Phenoflow analysis package in R (8).

***Bacterial amplicon sequencing***

To characterize the taxonomic composition of the inoculants (i.e., axenic, Pond 2 and Pond 3 filtrates) that were used to inoculate axenic phytoplankton communities with freshwater bacteria found in association with each of the five host species at the start of the experiment (T_0_), we collected biomass from ~500 mL of each inoculant onto 0.22 μm nitrocellulose filters, snap froze filters in liquid nitrogen, and stored at -80°C. Similarly, to characterize bacterial taxonomic composition at the end of the six-week experiment, we collected biomass onto 0.22 μm nitrocellulose filters, froze filters in liquid nitrogen, and stored at -80°C. For these T_f_ samples collected at the end of six weeks, we pooled our five biological replicates per treatment combination to obtain a total of 125 mL in volume per sample due to limited volume per flask. To collect this biomass from all five biological replicates onto a single filter, we poured each replicate into a sterile glass funnel on a vacuum manifold. For DNA extraction, all filters were then thawed and incubated for 60 minutes at 56°C in 30 μL proteinase K, 100 μL of ATL tissue lysis buffer and 300 μL AL lysis buffer sourced from Qiagen. Cells were then lysed by vortexing for 10 minutes and DNA was extracted and purified using a DNeasy Blood and Tissue Kit (Qiagen, Hilden, Germany). We then targeted the amplification of the V4 region of the 16S rRNA gene using the 515f/806r primer pair (9). The sequences for 515f and 806r are 5’-GTGYCAGCMGCCGCGGTAA-3’ and 5’-GGACTACNVGGGTWTCTAAT-3’, respectively (10, 11). Amplicon products were multiplexed with a unique 12 bp sequence per sample and then combined in an equal amount creating the final amplicon pool. Amplicons were then cleaned using the Qiagen UltraClean PCR Clean-Up Kit following manufacturer’s instructions (Qiagen, Hilden, Germany). The final pool of 16S rRNA amplicon products were sequenced on an Illumina MiSeq instrument set to read a PE 150 cycle at the UCSD Institute for Genomic Medicine (San Diego, California, United States).

Sequence reads were processed with the QIIME2 bioinformatics platform release 2020.8, which provides rapid, scalable and reproducible analysis of microbiome data (12). In brief, we demultiplexed our paired end libraries, quality filtered reads based on Q scores (--p-min-quality=4 and --p-quality-window=3), trimmed off forward and reverse primers, and then denoised and generated Amplicon Sequence Variants (ASVs) using the DADA2 plugin (13). All merged reads were assigned taxonomy using SILVA 138, a reference database for 16S rRNA genes (14, 15). ASVs were globally aligned using MAFFT v7 (16) and a phylogenetic tree was built using FastTree v2.1.4 and a GTR-CAT model of rate heterogeneity (17). The R package phyloseq v1.36.0 was used to combine the ASV table, metadata, assigned taxonomic data, and the phylogenetic tree into a single, flexible object in R v4.1.1 (18, 19). We removed all ASVs that were classified as “Eukaryotes” and “Unassigned” at the Kingdom level, “Chloroplast” at the Order level, and “Mitochondria” at the Family level using the subset_taxa function in the phyloseq package. Further, to facilitate accurate interpretation of low biomass samples, we removed all ASVs from our study samples that were found in either the four no-template-control blanks and the four mock microbial community standard controls, which were prepared by and included in our sequencing lane by the UCSD Microbiome Core. This removed 83 ASVs from our full 16S dataset that we attribute to contamination during library preparation and/or sequencing, which has been shown to commonly cause significant bias for low-biomass samples (20). This resulted in a 16S dataset with 171 total ASVs across 27 samples (24 experimental flask samples and the three T_0_ inoculants) with a median depth of 13,999 bacterial reads (i.e. after removing algal chloroplast reads). For statistical analyses comparing the effects of our three treatments on bacterial composition and diversity, we filtered out the T_0_ inoculants samples, which resulted in a dataset with 141 ASVs and a median bacterial read depth of 13,655 prior to rarefaction.

Lastly, ASV relative abundances were determined after rarefying using the rrarefy function in the vegan package. As would be expected, many of our samples in the low microbiome diversity treatment had exceedingly few sequence reads. Therefore, we rarefied two separate times with and without the low microbiome diversity treatment group. When retaining all three levels of microbiome diversity, we rarefied to 14 bacterial reads, whereas when retaining only the medium and high levels of microbiome diversity, we rarefied to 6,121 bacterial reads. We report mean ASV abundances for taxa found in each level of the microbiome diversity, phosphorus, and temperature treatments in Table S1. Using non-rarefied data, we created heat maps depicting change in log 10 abundance of all 171 ASVs recovered through this study using the comp_heatmap function in the microViz R package v. 0.12.4 (21). To quantify the percent contribution of potential source samples (e.g., T_0_ inoculants and experimental flasks) of sequences found in our low microbiome samples we used SourceTracker2 with default parameters (22)

***Phytoplankton community dynamics***

We tracked how phytoplankton cell counts varied by treatment over the duration of the study by preserving 1 mL from each flask with 5 μL of 25% glutaraldehyde and storing at 4°C. After weekly sampling, we also replaced 10% of the media with fresh media, abiding by the correct phosphorus-level media being added to each flask. We assessed phytoplankton cell density and species composition of all 120 experimental flasks at the end of the six-week study by counting either 400 cells or four hemocytometer grids (3.6 μL), whichever came first, of each phytoplankton species per sample (23). We also measured phytoplankton biomass at the end of the six-week study using the methods described below for stoichiometric analysis.

***Phytoplankton morphological analysis***

To determine how phytoplankton cell size varied by each unique treatment combination, two experimental flasks from each combination were randomly selected for analysis using image flow cytometry. From each flask, we preserved 1 mL of culture with 5 μL of 25% glutaraldehyde and stored at 4°C. Samples were processed using the Amnis ImageStream^X^ Mk II Flow Cytometer (Cytek Biosciences, Fremont, California, USA) with 60X objective in low speed/high sensitivity mode and 6-15 mW of 488 nm excitation. Acquisition stop criteria was set to 10 minutes or 2,000 events. Cell size data was then processed with Amnis AI Software, which uses random forest and convolution neural networks to score data into the five training classes, one per phytoplankton species in our phytoplankton communities. Amnis AI Software requires a minimum of 120 ground truth images per training class which were sourced from monocultures of *C. sorokiniana*, *C. microporum, S. acuminatus*, and *S. capricornutum.* However, for *M. minutum*, ground truth images were manually tagged using Amnis IDEAS Software from a single polyculture with known abundances of the five phytoplankton species. The following feature parameters were then measured per phytoplankton species per sample: cell area (μm^2^), diameter (μm), height (μm), and perimeter (μm).

***Ecosystem nutrient cycling***

At the end of the six-week study, total dissolved nitrogen and total dissolved phosphorous remaining in spent growth media was measured in each of the 120 flasks again using the Valderrama 1981 protocol (6). We pelleted phytoplankton and bacterial cells in 50 mL of each culture at 6000 g for 10 minutes, transferred 40 mL of the supernatant to pre-rinsed bottles, and stored immediately at -20°C until processing at the Marine Chemistry Lab.

***Statistical analysis***

To test whether bacterial phenotypic diversity, as measured via flow cytometry, varied across microbiome diversity treatments, we used our *a priori* ordered predictions to calculate a directional ANOVA using Spearman’s rank correlations (24). Using 16S amplicon data, we tested the relationship of bacterial community composition and phylogenetic membership with each experimental treatment. We first created a quantitative Jaccard and weighted UniFrac distance matrix from the rarefied ASV table. For each distance matrix, we implemented a distance-based redundancy analysis (db-RDA) for hypothesis testing. We then evaluated the statistical significance of each of the three main effects in our models: microbiome diversity treatment, phosphorus treatment and temperature treatment, using a permutational analysis of variance (permutations = 10,000) with the anova function in the stats package and the how function from the permute package (25, 26). Bacterial alpha diversity (e.g., ASV richness) was calculated with the d function from the vegetarian v.1.2 package for our T_0_ inoculants and each unique treatment combination sample that underwent amplicon sequencing. We used a multiple linear regression with bacterial alpha diversity predicted by our three treatments: microbiome diversity, phosphorus treatment, and temperature treatment, and an analysis of variance on that model for hypothesis testing (25, 26). Lastly, we illustrated the number of unique and shared common ASVs among the T_0_ inoculants and among the microbiome diversity treatments with a Venn diagram created by the function ggvenn within the R package ggvenn v.0.1.10 (27). Within each treatment group, proportional abundance per ASV was calculated by dividing the number of reads summed across samples by the total read depth within a treatment. ASVs within each group that had a proportional abundance <1%, and considered rare, were excluded from this comparison.

In addition, we then used three-way analysis of variance to evaluate the independent and interactive effects of our three treatments on each of the following dependent variables: δ^15^N, δ^13^C, cell abundance of each of our five phytoplankton species, Shannon’s diversity of the phytoplankton community (as calculated in the vegan package), total dissolved nitrogen and total dissolved phosphorus. To examine significantly different pairwise comparisons within each level of our nutrient treatment, we subset data by trophic status, ran an analysis of variance that evaluated the independent and interactive effects of microbiome diversity and temperature on each response variable, and then conducted Tukey’s HSD tests using the TukeyHSD function in the stats package (25, 26) Significance is denoted by a compact letter display in our main text figures. To measure the effects of our treatments on phytoplankton cell morphology, we used a multiple linear regression and multivariate analysis of variance approach (MANOVA) using the manova function from the car package in R (28). Specifically, we modeled the collective outcome of mean cell area, mean cell diameter, mean cell height, and the mean cell perimeter as predicted by the phosphorus, microbiome diversity, and temperature treatments with phytoplankton species as an additional fixed effect, and interactions between all fixed effects. We also ran separate MANOVAs for each of the five phytoplankton species with fixed and interaction effects for our three treatments.

**Supplemental Figures:**

**
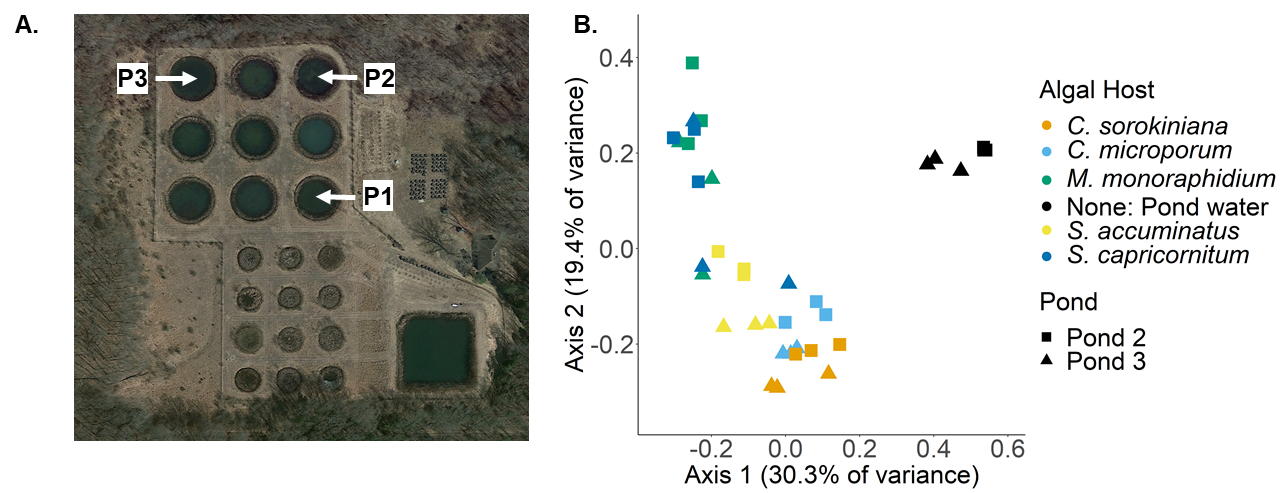
**

**Figure S1. A)** Aerial view of the naturalized experimental pond facility located at the University of Michigan E. S. George Reserve in Pinckney, MI, USA. **B)** As described in our prior work, the particle-associated bacterial communities inhabiting the water column of the two ponds used in this study were distinct (adonis: *F*_1,35_ = 11.7, p < 0.01, R^2^ = 0.13; Jackrel et al. 2020). Further, initially axenic phytoplankton hosts recruited a subset of bacterial taxa from these ponds and this recruitment was host-species specific (adonis: host - *F*_3,35_ = 10.5, p < 0.01, R^2^ = 0.56). In the present study, we expand on this work by using the same bacterial communities recruited by these five monocultures of phytoplankton from Pond 2 and Pond 3 to create our microbiome diversity treatments and to inoculate our axenic phytoplankton communities at the beginning of this study.

**
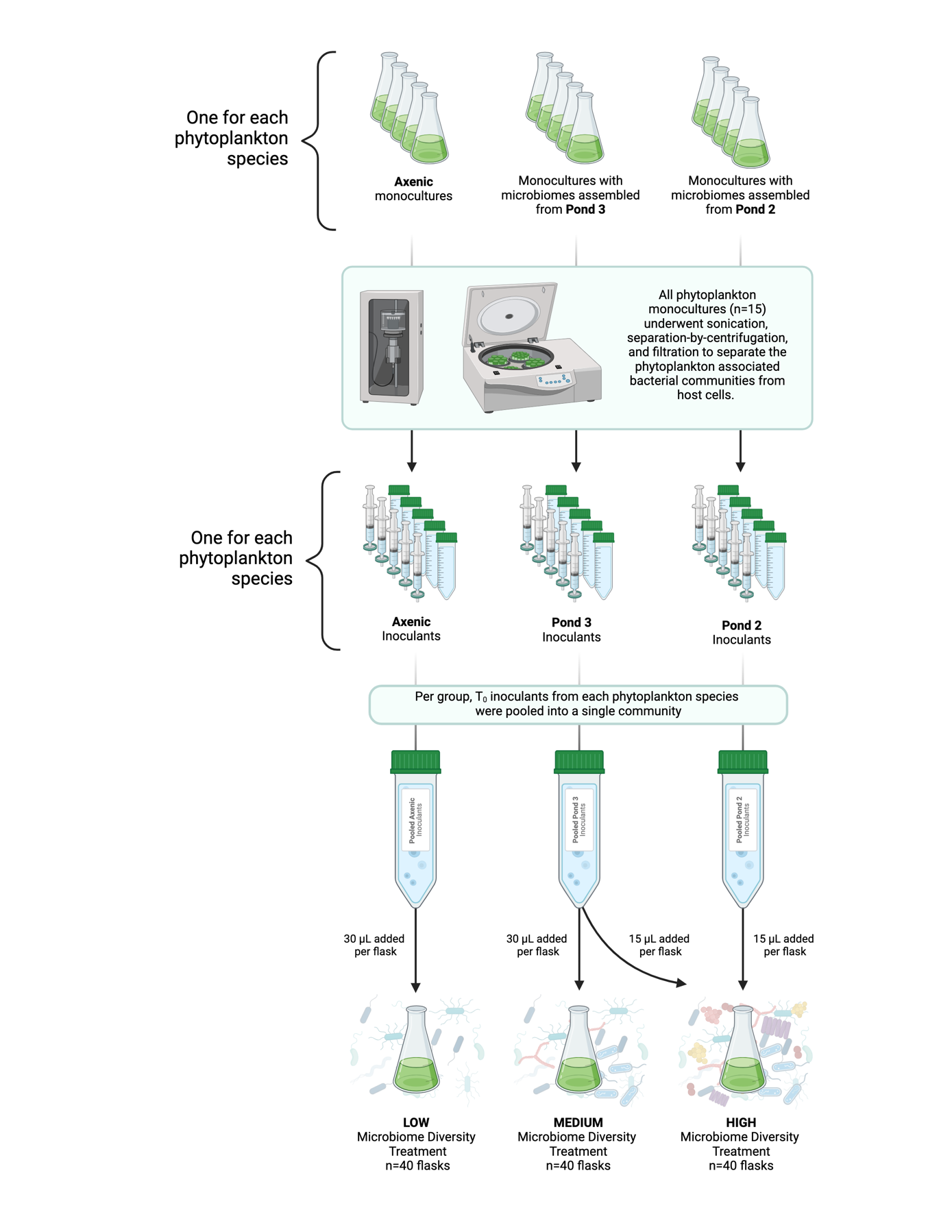
**

**Figure S2.** An illustration of the approach used to create the three levels of our microbiome diversity treatment that were then inoculated into the 120 flasks at the start of this experiment (T_0_). This illustration was created using BioRender.

**Figure S3.** Heat map illustrating bacterial log 10 abundance at the ASV level for each T_0_ inoculant containing Axenic, Pond 2, and Pond 3 bacterial communities that were used to create our microbiome diversity treatments, following Fig. S2. Columns correspond to each sample from our 16S rRNA data set, while rows correspond to bacterial families. The left side of the heatmap is flanked by a dendrogram depicting hierarchical clustering. Non-rarefied abundances were used and are represented in this figure due to low read depth within our axenic inoculant. Note that the axenic inoculant contained lower read depth and similar taxonomic richness than our no-template-control blanks (see Table S1), and was likely bacterial free. The sequence reads that do appear in our axenic inoculant are common genera found in human gut microbiomes (30) and are likely an artifact of sequencing low biomass samples on a shared instrument (20).

**Figure S4.** Venn diagram describing the shared number of ASVs and shared read depths among the three T_0_ inoculants that were used to create our microbiome diversity treatments, following Fig. S2. **A)** When common and rare ASVs were examined, 15 ASV and 74.27% of total reads were shared among the two inoculants, while the axenic inoculant shared no ASVs with Pond 2 or Pond 3 inoculants. **B)** When only common ASVs were examined (>1% in proportional abundance within an inoculant), the number of shared and unique ASVs substantially drop and only 4 ASVs were shared among the two pond inoculants with 60.92% of total reads shared.


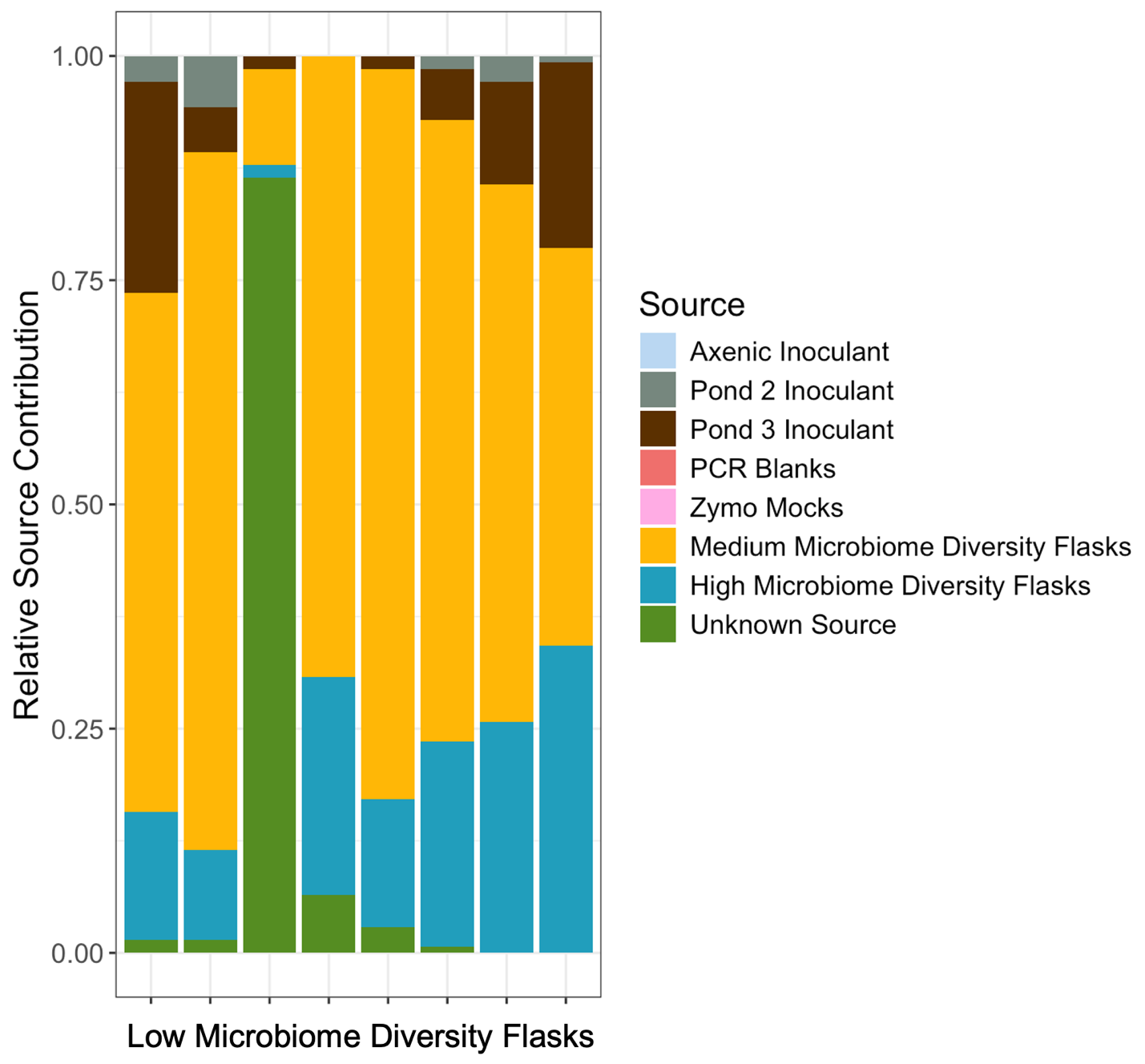


**Figure S5.** In our low microbiome diversity flasks (n = 8), medium and high microbiome diversity flasks accounted for, on average, 58.62% and 19.75% of source contamination according to Bayesian SourceTracker2. In addition, Pond 2 and Pond 3 inoculants accounted for 1.69% and 8.66% of source contamination, while the axenic inoculant, PCR blanks, and Zymo mock communities contributed 0% to low microbiome diversity flasks. We have determined that the unknown source of ASV found in the third flask from the left, which is our lowest biomass sample (read depth of 14) out of the low microbiome diversity flasks, is a common genus of the human gut microbiome, *Bifidobacterium*, and is likely an artifact from sequencing on a shared instrument. We have included it here for full transparency. See Table S2 for a full SourceTracker2 summary of the proportional contributions from source samples.


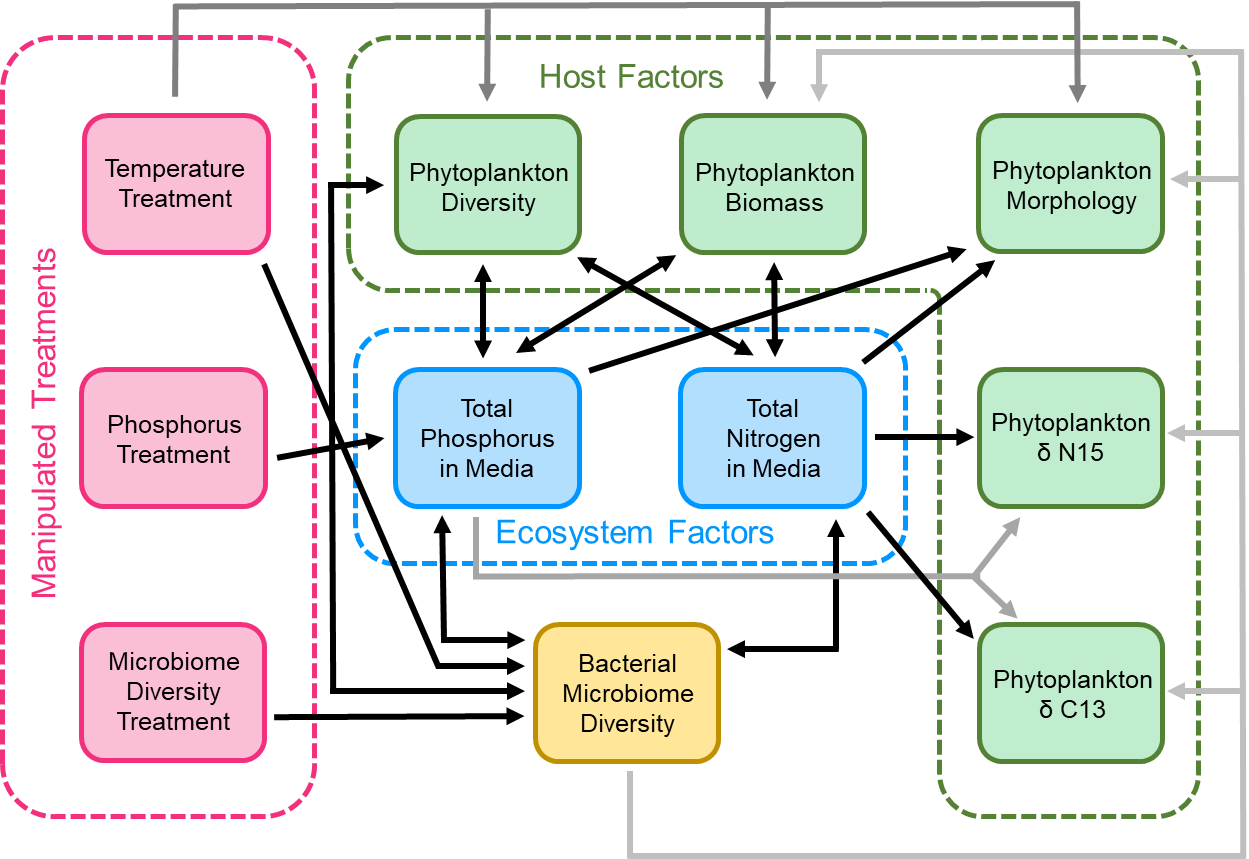


**Figure S6.** We have constructed a directed acyclic graph (DAG) illustrating hypothesized causal pathways from our three treatments, microbiome diversity, temperature, and phosphorus to ecosystem factors and host factors that we measured in this study. Single arrows are shown in black and branching arrows are shown in shades of gray. Double-headed arrows indicate bidirectional effects. For simplicity, we have combined phytoplankton biomass and cell density as “Phytoplankton Biomass” within this diagram as they represent a similar host factor. This conceptual diagram outlines direct causal paths that have influenced the way in which we model and interpret our results at the end of our six-week experiment. For example, our microbiome diversity treatment affects phytoplankton biomass, morphology, and phytoplankton δ^15^N and δ^13^C, but is conditional on bacterial microbiome diversity. Similarly, our DAG illustrates that by the end of our six-week study, the phosphorus treatment affected total nitrogen in media through the mediation by bacterial microbiome diversity. We can infer this as each experimental unit included in this study was a closed system and received the same starting concentration of nitrogen.

**Figure S7.** At the end of the six-week study, we detected significant differences in bacterial taxonomic diversity among our microbiome diversity treatments when amplicon data was **A)** not rarefied (*F*_2,17_=61.68, p<0.001), **B)** rarefied to the lowest number of reads among all three microbiome treatments (14 reads; *F*_2,17_=6.52, p<0.01), and **C)** rarefied to lowest number of reads between medium and high microbiome treatments (6,121 reads; *F*_1,10_=5.82, p<0.05).

**Figure S8.** Among medium and high microbiome diversity treatments, bacterial community composition was significantly influenced by the level of microbiome diversity and by phosphorous treatments, represented as colors and shapes respectively. Amplicon data represented in this figure was rarefied to the lowest number of bacterial reads among these two groups (6,121 reads). Two db-RDAs were built using the **A)** quantitative Jaccard and **B)** weighted UniFrac distance metric and both were analyzed with a permutational analysis of variance (10,000 permutations) to uncover the effects of our treatments on bacterial community composition and phylogenetic membership (quantitative Jaccard: microbiome diversity: *F*_1,10_=8.15, p<0.0001; phosphorus: *F*_3,10_=2.75, p<0.01; weighted UniFrac: microbiome diversity: *F*_1,10_=9.59, p<0.0001; phosphorus: *F*_3,10_=4.44, p<0.001). Our temperature treatment did not have an effect on microbiome community composition (quantitative Jaccard: *F*_1,10_=1.78, p>0.05; weighted UniFrac: *F*_1,10_=1.11, p>0.05).

**Figure S9.** Heat map illustrating bacterial log 10 abundance at the family level for each unique combination of our three treatments. Columns correspond to each sample from our 16S rRNA data set, while rows correspond to bacterial families. T_0_ inoculants containing Axenic, Pond 2, and Pond 3 bacterial communities that were used to create our microbiome diversity treatments, following Fig. S2, are represented as the three columns on the far right. The left side of the heatmap is flanked by a dendrogram depicting hierarchical clustering. Non-rarefied abundances were used and are represented in this figure due to low read depth within the low microbiome diversity treatment.

**Figure S10.** Taxonomic heat map illustrating change in bacterial log abundance at the ASV level across each unique treatment combination. Columns correspond to each sample from our 16S rRNA data set. T_0_ inoculants containing Axenic, Pond 2, and Pond 3 Bacterial Communities that were used to create our microbiome diversity treatments, following Fig. S2, are represented as the three columns on the far right. Taxonomic annotation on the right side of the heat map displays the bacterial family, genus, species, and ASV number from our study. If an ASV was unclassified at the family level, then only the ASV number is denoted as a row name. ASVs that are not classified at the Genus or species level display the bacterial family and ASV number. The dendrogram on the left of the heat map illustrates hierarchical clustering. Non-rarefied abundances were used and are represented in this figure due to low read depth within the low microbiome diversity treatment.


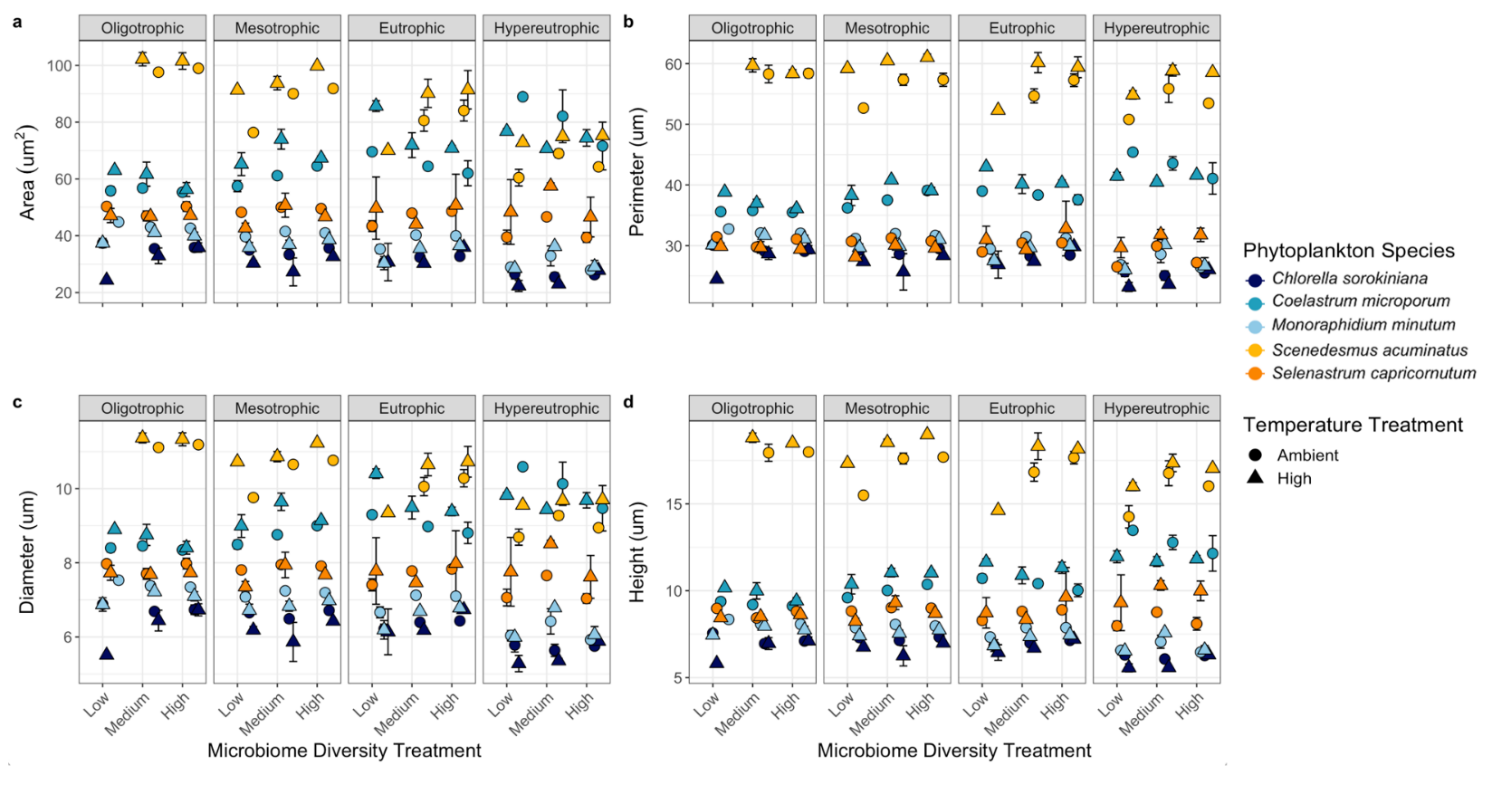


**Figure S11.** Cell morphological plasticity across our phosphorus, microbiome and temperature treatments quantified using imaging flow cytometry. The mean ± SE of **a)** cell area, **b)** cell perimeter, **c)** cell diameter, and **d)** cell height are reported for each of our five species of phytoplankton. Multiple analysis of variance tests were ran using all four of these metrics for the whole five-species community of phytoplankton and for each of the five species independently. MANOVA tables are reported as Tables S4 – S9.

**
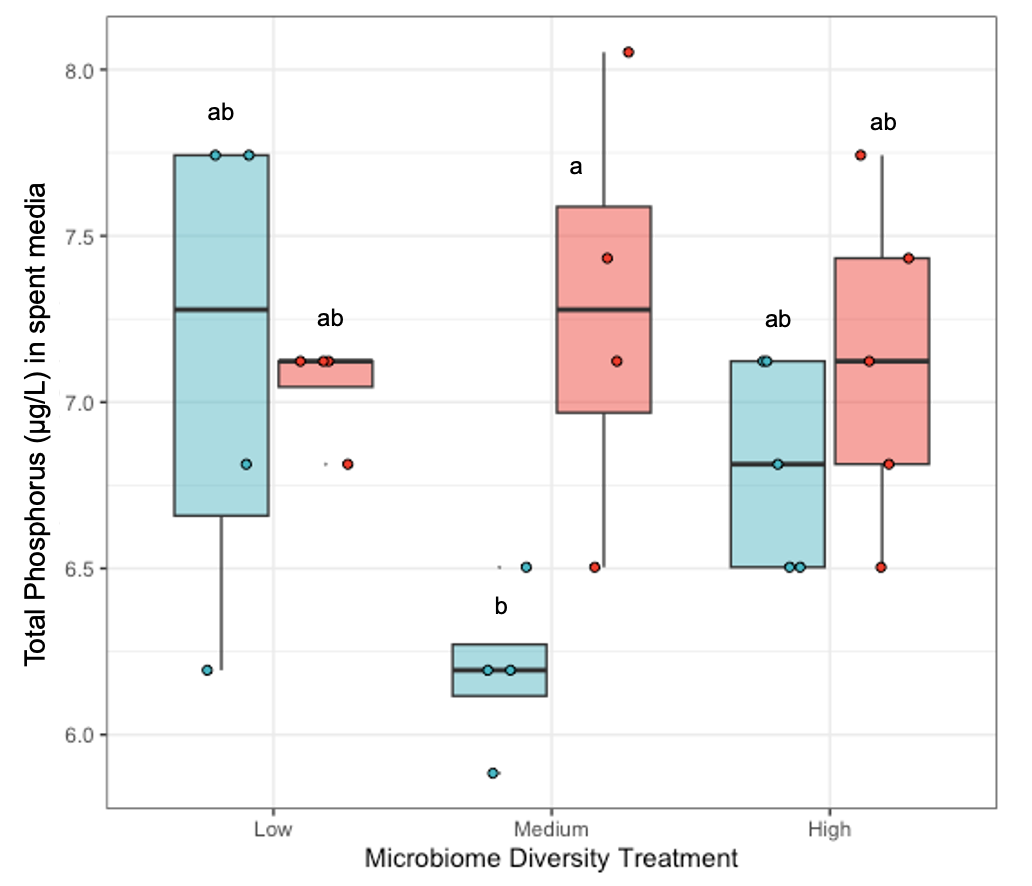
**

**Figure S12.** Trophic state, as determined by phosphorus concentration, was the dominant driver of total phosphorus in spent media, while microbiome diversity was a significant but secondary driver of total N and P concentrations at the end of the six-week study. Total P concentrations were measured of the supernatant from cultures that were pelleted to remove phytoplankton and bacterial cells. ANOVA total phosphorus - microbiome diversity: *F*_2,87_=12.45, *p*<0.0001, phosphorus: *F*_3,87_=173.25, *p*<0.0001, temperature: *F*_1,87_=0.12, *p=*0.73. See Table S18-S19 for ANOVA tables and main text Fig. 6 for results across all nutrient treatments. Pairwise comparisons were made within each level of the nutrient treatment, in this case the oligotrophic environment, and statistically similar comparisons are denoted by letters.

**Supplemental Tables:**

**Table S1.** A summary table describing ASV richness and bacterial read depth across our T_0_ samples (i.e., Axenic, Pond 2, and Pond 3 inoculants), four no-template-control PCR blanks, and the four-sample dilution series of a ZymoBiomic Microbial Community Standard that were included in our 16S rRNA sequencing run at UCSD Institute for Genomic Medicine.

|  | **ASV Richness** | **Read Depth** |
| --- | --- | --- |
| Axenic Inoculant | 14 | 147 |
| Pond 2 Inoculant | 37 | 18,874 |
| Pond 3 Inoculant | 42 | 19,245 |
| PCR Blank 1 | 14 | 489 |
| PCR Blank 2 | 12 | 360 |
| PCR Blank 3 | 11 | 557 |
| PCR Blank 4 | 20 | 869 |
| Zymo PCR 0.01 | 30 | 1,837 |
| Zymo PCR 0.1 | 10 | 16,169 |
| Zymo PCR 1.0 | 12 | 22,435 |
| Zymo PCR 10.0 | 8 | 19,406 |

**Table S2.** Summary table from SourceTracker2 with proportional ASV contribution from potential source samples (rows) to low microbiome diversity (MD) flasks (sink; columns).

**Table S3.** Mean relative abundance of each ASV for all experimental treatments. Rows are individual ASVs with taxonomic assignment from the SILVA 138 database. Non-rarefied abundances shown due to low read depth within the low microbiome diversity treatment.

**Table S4.** Three-way ANOVA for the mean δ^13^C of dried phytoplankton biomass of the five-species phytoplankton community at the end of the six-week experiment.

|  | **DF** | **Sum Sq** | **Mean Sq** | **F value** | **Pr (>F)** |
| --- | --- | --- | --- | --- | --- |
| Micro. Div. | 2 | 20.7 | 10.34 | 11.958 | **2.45E10^-5^** |
| P | 3 | 553 | 184.35 | 213.252 | **< 2E10^-16^** |
| T | 1 | 3.7 | 3.68 | 4.252 | **0.04205** |
| P*T | 3 | 2.3 | 0.78 | 0.898 | 0.44562 |
| P*Micro. Div. | 6 | 18.8 | 3.13 | 3.615 | **0.00292** |
| T*Micro. Div. | 2 | 3.1 | 1.54 | 1.783 | 0.17402 |
| P*T*Micro. Div. | 6 | 9.6 | 1.6 | 1.85 | 0.09802 |
| Residuals | 91 | 78.7 | 0.86 |  |  |

**Table S5.** Three-way ANOVA for the mean δ^15^N of dried phytoplankton biomass of the five-species phytoplankton community at the end of the six-week experiment.

|  | **DF** | **Sum Sq** | **Mean Sq** | **F value** | **Pr (>F)** |
| --- | --- | --- | --- | --- | --- |
| Micro. Div. | 2 | 49.2 | 24.61 | 25.751 | **1.37E10^-9^** |
| P | 3 | 598.6 | 199.52 | 208.767 | **< 2E10^-16^** |
| T | 1 | 52.2 | 52.22 | 54.637 | **6.83E10^-11^** |
| P*T | 3 | 16.8 | 5.6 | 5.861 | **0.00105** |
| P*Micro. Div. | 6 | 76.5 | 12.74 | 13.335 | **8.68E10^-11^** |
| T*Micro. Div. | 2 | 9 | 4.48 | 4.688 | **0.01155** |
| P*T*Micro. Div. | 6 | 7.2 | 1.19 | 1.249 | 0.28906 |
| Residuals | 91 | 87 | 0.96 |  |  |

**Table S6.** Multivariate Analysis of Variance examining the mean area, diameter, height, and perimeter of phytoplankton cells found within the five-species phytoplankton community at the end of the six-week experiment.

|  | **DF** | **Wilks' Λ** | **Approx F** | **Num DF** | **Den DF** | **Pr (>F)** |
| --- | --- | --- | --- | --- | --- | --- |
| Micro. Div. | 2 | 0.9168 | 2.15 | 8 | 388 | **0.03045** |
| P | 3 | 0.5662 | 10.26 | 12 | 513.57 | **< 2E-16** |
| T | 1 | 0.9115 | 4.71 | 4 | 194 | **1.20E-03** |
| Phytoplankton Species | 4 | 0.0007 | 353.34 | 16 | 593.32 | **< 2E-16** |
| P*T | 3 | 0.9012 | 1.72 | 12 | 513.57 | 0.05996 |
| P*Micro. Div. | 6 | 0.8752 | 1.1 | 24 | 678 | 0.33693 |
| T*Micro. Div. | 2 | 0.9716 | 0.7 | 8 | 388 | 0.68893 |
| P*T*Micro. Div. | 6 | 0.9277 | 0.61 | 24 | 678 | 0.92578 |

**Table S7.** Multivariate Analysis of Variance examining the mean area, diameter, height, and perimeter of *Chlorella sorokiniana* cells found within the five-species phytoplankton community at the end of the six-week experiment.

|  | **DF** | **Wilks' Λ** | **Approx F** | **Num DF** | **Den DF** | **Pr (>F)** |
| --- | --- | --- | --- | --- | --- | --- |
| Micro. Div. | 2 | 0.33548 | 3.2692 | 8 | 36 | **0.006664** |
| P | 3 | 0.05268 | 8.1536 | 12 | 47.915 | **4.72E-08** |
| T | 1 | 0.2456 | 13.8223 | 4 | 18 | **2.53E-05** |
| P*T | 3 | 0.32233 | 2.1325 | 12 | 47.915 | **0.032087** |
| P*Micro. Div. | 6 | 0.19181 | 1.6143 | 24 | 64.005 | 0.066264 |
| T*Micro. Div. | 2 | 0.45471 | 2.1733 | 8 | 36 | 0.053504 |
| P*T*Micro. Div. | 6 | 0.20638 | 1.5254 | 24 | 64.005 | 0.092099 |

**Table S8.** Multivariate Analysis of Variance examining the mean area, diameter, height, and perimeter of *Coelastrum microporum* cells found within the five-species phytoplankton community at the end of the six-week experiment.

|  | **DF** | **Wilks' Λ** | **Approx F** | **Num DF** | **Den DF** | **Pr (>F)** |
| --- | --- | --- | --- | --- | --- | --- |
| Micro. Div. | 2 | 0.65816 | 1.105 | 8 | 38 | 0.381509 |
| P | 3 | 0.05746 | 8.1901 | 12 | 50.561 | **2.91E-08** |
| T | 1 | 0.68837 | 2.1503 | 4 | 19 | 0.114045 |
| P*T | 3 | 0.17577 | 3.9152 | 12 | 50.561 | **0.000294** |
| P*Micro. Div. | 6 | 0.28214 | 1.2296 | 24 | 67.493 | 0.250082 |
| T*Micro. Div. | 2 | 0.64219 | 1.1778 | 8 | 38 | 0.337588 |
| P*T*Micro. Div. | 6 | 0.17933 | 1.3806 | 24 | 67.493 | 0.151427 |

**Table S9.** Multivariate Analysis of Variance examining the mean area, diameter, height, and perimeter of *Monoraphidium minutum* cells found within the five-species phytoplankton community at the end of the six-week experiment.

|  | **DF** | **Wilks' Λ** | **Approx F** | **Num DF** | **Den DF** | **Pr (>F)** |
| --- | --- | --- | --- | --- | --- | --- |
| Micro. Div. | 2 | 0.41471 | 2.7642 | 8 | 40 | **0.01568** |
| P | 3 | 0.05099 | 9.2214 | 12 | 53.207 | **2.92E-09** |
| T | 1 | 0.26345 | 13.9792 | 4 | 20 | **1.35E-05** |
| P*T | 3 | 0.40924 | 1.7812 | 12 | 53.207 | 0.07557 |
| P*Micro. Div. | 6 | 0.20634 | 1.194 | 24 | 70.982 | 0.27737 |
| T*Micro. Div. | 2 | 0.71357 | 0.9191 | 8 | 40 | 0.5111 |
| P*T*Micro. Div. | 6 | 0.4547 | 0.7496 | 24 | 70.982 | 0.78278 |

**Table S10.** Multivariate Analysis of Variance examining the mean area, diameter, height, and perimeter of *Scenedesmus acuminatus* cells found within the five-species phytoplankton community at the end of the six-week experiment. Since all equal factorial comparisons could not be made within *S. acuminatus*, interaction effects were excluded from the multiple linear regression to avoid singularities.

|  | **DF** | **Wilks' Λ** | **Approx F** | **Num DF** | **Den DF** | **Pr (>F)** |
| --- | --- | --- | --- | --- | --- | --- |
| Micro. Div. | 2 | 0.2884 | 6.2501 | 8 | 58 | **8.19E-06** |
| P | 3 | 0.06677 | 11.4332 | 12 | 77.018 | **9.25E-13** |
| T | 1 | 0.4064 | 10.5894 | 4 | 29 | **2.53E-05** |

**Table S11.** Multivariate Analysis of Variance examining the mean area, diameter, height, and perimeter of *Selenastrum capricornutum* cells found within the five-species phytoplankton community at the end of the six-week experiment.

|  | **DF** | **Wilks' Λ** | **Approx F** | **Num DF** | **Den DF** | **Pr (>F)** |
| --- | --- | --- | --- | --- | --- | --- |
| Micro. Div. | 2 | 0.44241 | 2.6431 | 8 | 42 | **0.019188** |
| P | 3 | 0.7624 | 7.6583 | 12 | 55.852 | **3.87E-08** |
| T | 1 | 0.30169 | 12.152 | 4 | 21 | **2.86E-05** |
| P*T | 3 | 0.17349 | 4.3695 | 12 | 55.852 | **7.07E-05** |
| P*Micro. Div. | 6 | 0.09428 | 3.0031 | 24 | 74.47 | **0.000155** |
| T*Micro. Div. | 2 | 0.39908 | 3.0606 | 8 | 42 | **0.008319** |
| P*T*Micro. Div. | 6 | 0.13244 | 2.4362 | 24 | 74.47 | **0.001861** |

**Table S12.** Three-way ANOVA for mean dried phytoplankton biomass of the five-species phytoplankton community at the end of the six-week experiment.

|  | **DF** | **Sum Sq** | **Mean Sq** | **F value** | **Pr (>F)** |
| --- | --- | --- | --- | --- | --- |
| Micro. Div. | 2 | 6.268E10^-5^ | 3.134E10^-5^ | 51.982 | **6.27E10^-16^** |
| P | 3 | 6.970E10^-6^ | 2.324E10^-6^ | 3.855 | **0.0119** |
| T | 1 | 1.490E10^-6^ | 1.487E10^-6^ | 2.467 | 0.1196 |
| P*T | 3 | 2.040E10^-6^ | 6.810E10^-7^ | 1.129 | 0.3413 |
| P*Micro. Div. | 6 | 2.579E10^-5^ | 4.298E10^-6^ | 7.129 | **2.79E10^-6^** |
| T*Micro. Div. | 2 | 6.200E10^-7^ | 3.090E10^-7^ | 0.512 | 0.6009 |
| P*T*Micro. Div. | 6 | 2.510E10^-6^ | 4.180E10^-7^ | 0.694 | 0.6549 |
| Residuals | 94 | 5.667E10^-5^ | 6.030E10^-7^ |  |  |

**Table S13.** Three-way ANOVA for the mean log total phytoplankton cell density of the five-species phytoplankton community at the end of the six-week experiment.

|  | **DF** | **Sum Sq** | **Mean Sq** | **F value** | **Pr (>F)** |
| --- | --- | --- | --- | --- | --- |
| Micro. Div. | 2 | 0.358 | 0.179 | 3.65 | **0.029657** |
| P | 3 | 21.178 | 7.059 | 144.134 | **< 2E10^-16^** |
| T | 1 | 2.92 | 2.92 | 59.613 | **1.1E10^-11^** |
| P*T | 3 | 1.132 | 0.377 | 7.704 | **0.000115** |
| P*Micro. Div. | 6 | 1.074 | 0.179 | 3.655 | **0.002601** |
| T*Micro. Div. | 2 | 0.009 | 0.004 | 0.087 | 0.916949 |
| P*T*Micro. Div. | 6 | 0.213 | 0.035 | 0.724 | 0.631394 |
| Residuals | 96 | 4.702 | 0.049 |  |  |

**Table S14.** Three-way ANOVA for the mean Shannon Diversity of the five-species phytoplankton community at the end of the six-week experiment.

|  | **DF** | **Sum Sq** | **Mean Sq** | **F value** | **Pr (>F)** |
| --- | --- | --- | --- | --- | --- |
| Micro. Div. | 2 | 0.29 | 0.1449 | 4.204 | **0.01778** |
| P | 3 | 4.612 | 1.5373 | 44.596 | **< 2E10^-16^** |
| T | 1 | 0.001 | 0.0012 | 0.035 | 0.85136 |
| P*T | 3 | 0.271 | 0.0904 | 2.623 | 0.05497 |
| P*Micro. Div. | 6 | 0.614 | 0.1023 | 2.967 | **0.01061** |
| T*Micro. Div. | 2 | 0.41 | 0.2048 | 5.941 | **0.00369** |
| P*T*Micro. Div. | 6 | 0.429 | 0.0716 | 2.077 | 0.063 |
| Residuals | 96 | 3.309 | 0.0345 |  |  |

**Table S15.** Three-way ANOVA for the mean log cellular abundance of *Chlorella sorokiniana* at the end of the six-week experiment.

|  | **DF** | **Sum Sq** | **Mean Sq** | **F value** | **Pr (>F)** |
| --- | --- | --- | --- | --- | --- |
| Micro. Div. | 2 | 79.92 | 39.96 | 105.451 | **< 2E10^-16^** |
| P | 3 | 21.41 | 7.14 | 18.829 | **1.21E10^-9^** |
| T | 1 | 2.24 | 2.24 | 5.918 | **0.01688** |
| P*T | 3 | 5.15 | 1.72 | 4.529 | **0.00519** |
| P*Micro. Div. | 6 | 3.66 | 0.61 | 1.609 | 0.15312 |
| T*Micro. Div. | 2 | 0.42 | 0.21 | 0.548 | 0.58012 |
| P*T*Micro. Div. | 6 | 7.57 | 1.26 | 3.329 | **0.00512** |
| Residuals | 94 | 35.62 | 0.38 |  |  |

**Table S16.** Three-way ANOVA for the mean log cellular abundance of *Coelastrum microporum* at the end of the six-week experiment.

|  | **DF** | **Sum Sq** | **Mean Sq** | **F value** | **Pr (>F)** |
| --- | --- | --- | --- | --- | --- |
| Micro. Div. | 2 | 2.92 | 1.459 | 4.184 | **0.018101** |
| P | 3 | 59.25 | 19.749 | 56.653 | **< 2E10^-16^** |
| T | 1 | 3.7 | 3.702 | 10.62 | **0.001548** |
| P*T | 3 | 0.01 | 0.003 | 0.008 | 0.99904 |
| P*Micro. Div. | 6 | 8.87 | 1.478 | 4.24 | **0.000789** |
| T*Micro. Div. | 2 | 0.04 | 0.018 | 0.052 | 0.949126 |
| P*T*Micro. Div. | 6 | 3.98 | 0.664 | 1.904 | 0.087993 |
| Residuals | 96 | 33.47 | 0.349 |  |  |

**Table S17.** Three-way ANOVA for the mean log cellular abundance of *Monoraphidium minutum* at the end of the six-week experiment.

|  | **DF** | **Sum Sq** | **Mean Sq** | **F value** | **Pr (>F)** |
| --- | --- | --- | --- | --- | --- |
| Micro. Div. | 2 | 0.98 | 0.49 | 1.351 | 0.2638 |
| P | 3 | 0.03 | 0.01 | 0.029 | 0.99342 |
| T | 1 | 33.78 | 33.78 | 93.055 | **8.55E10^-16^** |
| P*T | 3 | 3.61 | 1.2 | 3.314 | **0.02324** |
| P*Micro. Div. | 6 | 7.83 | 1.31 | 3.595 | **0.00294** |
| T*Micro. Div. | 2 | 0.5 | 0.25 | 0.684 | 0.50718 |
| P*T*Micro. Div. | 6 | 8.47 | 1.41 | 3.89 | **0.00161** |
| Residuals | 96 |  |  |  |  |

**Table S18.** Three-way ANOVA for the mean log cellular abundance of *Scenedesmus acuminatus* at the end of the six-week experiment.

|  | **DF** | **Sum Sq** | **Mean Sq** | **F value** | **Pr (>F)** |
| --- | --- | --- | --- | --- | --- |
| Micro. Div. | 2 | 3.03 | 1.51 | 1.692 | 0.189592 |
| P | 3 | 195.64 | 65.21 | 72.96 | **< 2E10^-16^** |
| T | 1 | 11.59 | 11.59 | 12.962 | **0.000508** |
| P*T | 3 | 0.72 | 0.24 | 0.27 | 0.846723 |
| P*Micro. Div. | 6 | 25.29 | 4.21 | 4.715 | **0.000304** |
| T*Micro. Div. | 2 | 0.26 | 0.13 | 0.144 | 0.865845 |
| P*T*Micro. Div. | 6 | 4.18 | 0.7 | 0.78 | 0.587681 |
| Residuals | 95 | 84.91 | 0.89 |  |  |

**Table S19.** Three-way ANOVA for the mean log cellular abundance of *Selenastrum capricornutum* at the end of the six-week experiment.

|  | **DF** | **Sum Sq** | **Mean Sq** | **F value** | **Pr (>F)** |
| --- | --- | --- | --- | --- | --- |
| Micro. Div. | 2 | 16.44 | 8.22 | 11.252 | **4.62E10^-5^** |
| P | 3 | 4.63 | 1.54 | 2.112 | 0.104666 |
| T | 1 | 45.98 | 45.98 | 62.954 | **7.67E10^-12^** |
| P*T | 3 | 7.85 | 2.62 | 3.584 | **0.017069** |
| P*Micro. Div. | 6 | 10.74 | 1.79 | 2.451 | **0.031071** |
| T*Micro. Div. | 2 | 13.56 | 6.78 | 9.281 | **0.000226** |
| P*T*Micro. Div. | 6 | 4.24 | 0.71 | 0.967 | 0.452805 |
| Residuals | 85 | 62.08 | 0.73 |  |  |

**Table S20.** Three-way ANOVA for mean total nitrogen in the phytoplankton media at the end of the six-week experiment.

|  | **DF** | **Sum Sq** | **Mean Sq** | **F value** | **Pr (>F)** |
| --- | --- | --- | --- | --- | --- |
| Micro. Div. | 2 | 580050 | 290025 | 14.745 | **2.91E10^-6^** |
| P | 3 | 7843724 | 2614575 | 132.931 | **< 2E10^-16^** |
| T | 1 | 43799 | 43799 | 2.227 | 0.139248 |
| P*T | 3 | 262701 | 87567 | 4.452 | **0.005865** |
| P*Micro. Div. | 6 | 494709 | 82451 | 4.192 | **0.000947** |
| T*Micro. Div. | 2 | 28868 | 14434 | 0.734 | 0.483001 |
| P*T*Micro. Div. | 6 | 104378 | 17396 | 0.884 | 0.509906 |
| Residuals | 87 | 1711179 | 19669 |  |  |

**Table S21.** Three-way ANOVA for mean total phosphorus in the phytoplankton media at the end of the six-week experiment.

|  | **DF** | **Sum Sq** | **Mean Sq** | **F value** | **Pr (>F)** |
| --- | --- | --- | --- | --- | --- |
| Micro. Div. | 2 | 2.47 | 1.234 | 12.452 | **1.75E10^-5^** |
| P | 3 | 51.50 | 17.167 | 173.25 | **< 2E10^-16^** |
| T | 1 | 0.01 | 0.012 | 0.119 | 0.73116 |
| P*T | 3 | 0.64 | 0.213 | 2.153 | 0.09932 |
| P*Micro. Div. | 6 | 1.90 | 0.317 | 3.197 | **0.00696** |
| T*Micro. Div. | 2 | 0.35 | 0.175 | 1.762 | 0.17770 |
| P*T*Micro. Div. | 6 | 0.86 | 0.144 | 1.449 | 0.20557 |
| Residuals | 87 | 8.62 | 0.099 |  |  |

**Supplemental References**

1. Jackrel SL, Schmidt KC, Cardinale BJ, Denef VJ. 2020. Microbiomes reduce their host’s sensitivity to interspecific interactions. mBio 11.

2. Jackrel SL, Yang JW, Schmidt KC, Denef VJ. 2021. Host specificity of microbiome assembly and its fitness effects in phytoplankton. ISME Journal 15:774–788.

3. Werner EE, McPeek MA. 1994. Direct and indirect effects of predators on two anuran species along an environmental gradient. Ecology 75:1368–1382.

4. Kilham SS, Kreeger DA, Lynn SG, Goulden CE, Herrera L. 1998. COMBO: A defined freshwater culture medium for algae and zooplankton. Hydrobiologia 377:147–159.

5. Seymour JR, Amin SA, Raina JB, Stocker R. 2017. Zooming in on the phycosphere: The ecological interface for phytoplankton-bacteria relationships. Nat Microbiol 2.

6. Valderrama JC. 1981. The simultaneous analysis of total nitrogen and total phosphorus in natural waters. Mar Chem 10:109–122.

7. Soranno PA, Bacon LC, Beauchene M, Bednar KE, Bissell EG, Boudreau CK, Boyer MG, Bremigan MT, Carpenter SR, Carr JW, Cheruvelil KS, Christel ST, Claucherty M, Collins SM, Conroy JD, Downing JA, Dukett J, Fergus CE, Filstrup CT, Funk C, Gonzalez MJ, Green LT, Gries C, Halfman JD, Hamilton SK, Hanson PC, Henry EN, Herron EM, Hockings C, Jackson JR, Jacobson-Hedin K, Janus LL, Jones WW, Jones JR, Keson CM, King KBS, Kishbaugh SA, Lapierre JF, Lathrop B, Latimore JA, Lee Y, Lottig NR, Lynch JA, Matthews LJ, McDowell WH, Moore KEB, Neff BP, Nelson SJ, Oliver SK, Pace ML, Pierson DC, Poisson AC, Pollard AI, Post DM, Reyes PO, Rosenberry DO, Roy KM, Rudstam LG, Sarnelle O, Schuldt NJ, Scott CE, Skaff NK, Smith NJ, Spinelli NR, Stachelek JJ, Stanley EH, Stoddard JL, Stopyak SB, Stow CA, Tallant JM, Tan PN, Thorpe AP, Vanni MJ, Wagner T, Watkins G, Weathers KC, Webster KE, White JD, Wilmes MK, Yuan S. 2017. LAGOS-NE: A multi-scaled geospatial and temporal database of lake ecological context and water quality for thousands of US lakes. Gigascience 6:1–22.

8. Props R, Monsieurs P, Mysara M, Clement L, Boon N. 2016. Measuring the biodiversity of microbial communities by flow cytometry. Methods Ecol Evol 7:1376–1385.

9. Walters W, Hyde ER, Berg-lyons D, Ackermann G, Humphrey G, Parada A, Gilbert J a, Jansson JK. 2015. Improved Bacterial 16S rRNA Gene (V4 and V4-5) and Fungal Internal Transcribed Spacer Marker Gene Primers for Microbial Community Surveys. mSystems 1:e0009-15.

10. Parada AE, Needham DM, Fuhrman JA. 2016. Every base matters: Assessing small subunit rRNA primers for marine microbiomes with mock communities, time series and global field samples. Environ Microbiol 18:1403–1414.

11. Apprill A, Mcnally S, Parsons R, Weber L. 2015. Minor revision to V4 region SSU rRNA 806R gene primer greatly increases detection of SAR11 bacterioplankton. Aquatic Microbial Ecology 75:129–137.

12. Bolyen E, Rideout JR, Dillon MR, Bokulich NA, Abnet CC, Al-Ghalith GA, Alexander H, Alm EJ, Arumugam M, Asnicar F, Bai Y, Bisanz JE, Bittinger K, Brejnrod A, Brislawn CJ, Brown CT, Callahan BJ, Caraballo-Rodríguez AM, Chase J, Cope EK, Da Silva R, Diener C, Dorrestein PC, Douglas GM, Durall DM, Duvallet C, Edwardson CF, Ernst M, Estaki M, Fouquier J, Gauglitz JM, Gibbons SM, Gibson DL, Gonzalez A, Gorlick K, Guo J, Hillmann B, Holmes S, Holste H, Huttenhower C, Huttley GA, Janssen S, Jarmusch AK, Jiang L, Kaehler BD, Kang K Bin, Keefe CR, Keim P, Kelley ST, Knights D, Koester I, Kosciolek T, Kreps J, Langille MGI, Lee J, Ley R, Liu YX, Loftfield E, Lozupone C, Maher M, Marotz C, Martin BD, McDonald D, McIver LJ, Melnik A V., Metcalf JL, Morgan SC, Morton JT, Naimey AT, Navas-Molina JA, Nothias LF, Orchanian SB, Pearson T, Peoples SL, Petras D, Preuss ML, Pruesse E, Rasmussen LB, Rivers A, Robeson MS, Rosenthal P, Segata N, Shaffer M, Shiffer A, Sinha R, Song SJ, Spear JR, Swafford AD, Thompson LR, Torres PJ, Trinh P, Tripathi A, Turnbaugh PJ, Ul-Hasan S, van der Hooft JJJ, Vargas F, Vázquez-Baeza Y, Vogtmann E, von Hippel M, Walters W, Wan Y, Wang M, Warren J, Weber KC, Williamson CHD, Willis AD, Xu ZZ, Zaneveld JR, Zhang Y, Zhu Q, Knight R, Caporaso JG. 2019. Reproducible, interactive, scalable and extensible microbiome data science using QIIME 2. Nat Biotechnol 37:852–857.

13. Callahan BJ, McMurdie PJ, Rosen MJ, Han AW, Johnson AJA, Holmes SP. 2016. DADA2: High-resolution sample inference from Illumina amplicon data. Nat Methods 13:581–583.

14. Pruesse E, Quast C, Knittel K, Fuchs BM, Ludwig W, Peplies J, Glöckner FO. 2007. SILVA: A comprehensive online resource for quality checked and aligned ribosomal RNA sequence data compatible with ARB. Nucleic Acids Res 35:7188–7196.

15. Quast C, Pruesse E, Yilmaz P, Gerken J, Schweer T, Yarza P, Peplies J, Glöckner FO. 2013. The SILVA ribosomal RNA gene database project: Improved data processing and web-based tools. Nucleic Acids Res 41:590–596.

16. Katoh K, Standley DM. 2013. MAFFT multiple sequence alignment software version 7: Improvements in performance and usability. Mol Biol Evol 30:772–780.

17. Price MN, Dehal PS, Arkin AP. 2010. FastTree 2 - Approximately maximum-likelihood trees for large alignments. PLoS One 5.

18. R Core Team A, Team RC. 2022. R: A language and environment for statistical computing. R Foundation for Statistical Computing, Vienna, Austria. 2012.

19. McMurdie PJ, Holmes S. 2013. Phyloseq: An R Package for Reproducible Interactive Analysis and Graphics of Microbiome Census Data. PLoS One 8.

20. Karstens L, Asquith M, Davin S, Fair D, Gregory WT, Wolfe AJ, Braun J, Mcweeney S. 2019. Controlling for Contaminants in Low-Biomass 16S rRNA Gene Sequencing Experiments. mSystems 4:1–14.

21. Barnett D, Arts I, Penders J. 2021. microViz: an R package for microbiome data visualization and statistics. J Open Source Softw 6:3201.

22. Knights D, Kuczynski J, Charlson ES, Zaneveld J, Mozer MC, Collman RG, Bushman FD, Knight R, Kelley ST. 2011. Bayesian community-wide culture-independent microbial source tracking. Nat Methods 8:761–765.

23. Narwani A, Lashaway AR, Hietala DC, Savage PE, Cardinale BJ. 2016. Power of Plankton: Effects of Algal Biodiversity on Biocrude Production and Stability. Environ Sci Technol 50:13142–13150.

24. Rice WR, Gaines SD. 1994. Extending nondirectional heterogeneity tests to evaluate simply ordered alternative hypotheses. Proc Natl Acad Sci U S A 91:225–226.

25. Team RC. 2018. Package “stats.” The R Stats Package 1–3.

26. Simpson GL, Team RC, Bates DM, Oksanen J, Simpson MGL. 2022. Package ‘permute.’

27. Yan L, Yan ML. 2021. Package “ggvenn.” CRAN.

28. Fox J, Weisberg S. 2018. An R companion to applied regression. Sage publications.

29. Kimmel K, Dee LE, Avolio ML, Ferraro PJ. 2021. Causal assumptions and causal inference in ecological experiments. Trends Ecol Evol 36:1141–1152.

30. Martino C, Dilmore AH, Burcham ZM, Metcalf JL, Jeste D, Knight R. 2022. Microbiota succession throughout life from the cradle to the grave. Nat Rev Microbiol 20:707–720.
